# Diversity, Function and Activity of DNA Viruses in the Qiangyong Proglacial Lake Sediment, the Tibetan Plateau

**DOI:** 10.1111/1758-2229.70262

**Published:** 2026-01-08

**Authors:** Yang Zhao, Meiling Feng, Hongfei Chi, Keshao Liu, Rong Wen, Weizhen Zhang, Pengfei Liu

**Affiliations:** ^1^ College of Ecology, Lanzhou University Lanzhou China; ^2^ Center for Pan‐Third Pole Environment, Lanzhou University Lanzhou China; ^3^ Key Laboratory of Pan‐Third Pole Biogeochemical Cycling Lanzhou China; ^4^ Chayu Integrated Observation and Research Station of the Xizang Autonomous Region Xizang China; ^5^ State Key Laboratory of Tibetan Plateau Earth System, Environment and Resources (TPESER) Institute of Tibetan Plateau Research, Chinese Academy of Sciences Beijing China; ^6^ University of Chinese Academy of Sciences Beijing China

**Keywords:** auxiliary metabolic gene, biogeochemical cycles, DNA viruses, proglacial lake sediments, the Tibetan plateau

## Abstract

Viruses are the most abundant biological entities on Earth and play crucial roles in regulating ecosystem processes and biogeochemical cycling. Proglacial lakes—key components of cryosphere aquatic systems—host diverse microbial communities despite extreme environmental conditions. However, the composition and ecological roles of DNA viral communities in proglacial lake sediments remain poorly understood. In this study, we applied metagenomic and metatranscriptomic approaches to investigate the diversity, function, activity and host interactions of DNA viruses in sediments from Qiangyong proglacial lake on the Tibetan Plateau. We recovered 4039 viral operational taxonomic units (vOTUs), with 76.6% unclassified at the family level, highlighting a vast reservoir of uncharacterized viral lineages. Host prediction linked 1.8% of vOTUs to key microbial taxa involved in carbon, nitrogen and sulphur cycling. We identified a broad array of virus‐encoded auxiliary metabolic genes (AMGs) involved in host resource utilization and metabolic transformation. Moreover, 63 AMGs not previously reported in the literature were discovered, significantly expanding the known viral functional gene repertoire. These findings offer new insights into the diversity and ecological potential of sediment‐associated DNA viruses in proglacial lakes, and emphasize their possible roles in shaping microbial communities and influencing biogeochemical processes in cold‐region ecosystems.

## Graphical abstract

This study identified DNA viruses from Qiangyong proglacial lake sediments on the Tibetan Plateau, revealing a highly diverse and previously uncharacterized viral community. These viruses influence microbial community structure and function by infecting key nutrient‐transforming taxa and encoding auxiliary metabolic genes that modulate host metabolism. Our findings highlight the overlooked ecological significance of sediment‐associated DNA viruses in proglacial lakes and underscore their potential roles in driving microbial interactions and biogeochemical cycling in high alpine aquatic ecosystems.

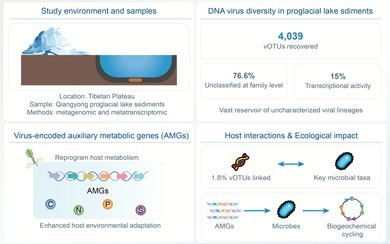

## 1 Introduction

Viruses are the most abundant biological entities on Earth and play a critical role in ecosystems (Dion et al. 2020; Breitbart 2012). They influence global biogeochemical cycling through close interactions with their hosts. Viruses can reshape microbial communities by infecting and lysing host cells, thereby altering the abundance and structure of functional microbes (Emerson et al. 2018; Braga et al. 2020). This phenomenon has been extensively studied in oceanic systems, where viruses eliminate 20%–40% of marine bacterial cells each day through lysis (Suttle 1994), thereby exerting top‐down control that restructures microbial community composition and evolutionary selection pressures, directly influencing carbon, nitrogen and sulphur cycling (Breitbart et al. 2018; Brum et al. 2016). This viral ‘shunt’” mechanism alters functional microbe abundance and creates ecological niches through species‐specific mortality, ultimately reprogramming microbial metabolic networks that govern global elemental cycling (Breitbart 2012; Roux et al. 2016). Viruses can also acquire host‐derived auxiliary metabolic genes (AMGs) via horizontal gene transfer. These AMGs enable viral manipulation of core host metabolic pathways, including photosynthesis, nucleotide synthesis and biogeochemical cycling of nitrogen, phosphorus and sulphur (Tian et al. 2024; Luo et al. 2022). By expressing AMG‐encoded enzymes during infection, viruses not only redirect host resources for replication but also expand environmental niche adaptation by conferring new metabolic functions (Breitbart et al. 2007; Thompson et al. 2011), effectively coupling viral reproduction strategies with biogeochemical transformations.

Proglacial lakes—water bodies that form at glacier termini—are an integral component of the cryosphere (Carrivick and Tweed 2013) and are increasingly prominent due to climate‐driven glacial retreat. The Tibetan Plateau (TP), known as the ‘Asian Water Tower’ (Yao et al. 2022), harbours the world's most extensive system of mid‐latitude glacial lakes, comprising over 15,800 lakes (> 0.02 km^2^) spanning more than 2250 km^2^ (Zhang et al. 2022, 2024). Climate‐induced glacial retreat has accelerated the expansion of these lakes by over 18% between 1990 and 2022 (Zhang, Wang, et al. 2023; Wang et al. 2020). Fed primarily by glacier meltwater, these lakes are marked by extreme environmental conditions, including persistently low temperatures, oligotrophy, high turbidity and elevated ultraviolet radiation (Moser et al. 2019; Marsh et al. 2024). Despite such harsh conditions, proglacial lakes host diverse microbial life, including bacteria, archaea, diatoms, zooplankton and select invertebrates (Marsh et al. 2024; Kleinteich et al. 2022; Liu et al. 2024; Liu, Liu, et al. 2017). These microorganisms underpin essential ecosystem processes: autotrophs such as cyanobacteria and algae fix carbon, while heterotrophs transform glacier‐derived organic matter (Sommaruga 2015; Kammerlander et al. 2016; Vanderwall et al. 2024). Recent studies also highlight their involvement in climate‐relevant processes, including methane production and oxidation (Wei et al. 2023; Huang et al. 2025), as well as the transformation of mercury from glacier meltwater (Mu et al. 2025).

Although microbial communities in proglacial lakes have received growing attention, viruses—key regulators of microbial dynamics—remain severely understudied in these systems. To date, only two studies have addressed viral ecology in proglacial lakes. Drewes et al. reported that viral abundance correlates with microbial abundance and glacial proximity (Drewes et al. 2016), while Liu et al. identified diverse RNA viruses, predominantly infecting prokaryotes, using metatranscriptomics (Liu et al. 2025). However, DNA viral communities, particularly in proglacial lake sediments where microbial activity is concentrated, have not been explored. This represents a critical gap in our understanding of viral diversity and function in high‐altitude aquatic ecosystems. Given the unique environmental pressures associated with glacial meltwater input and cryosphere extremes, we hypothesize that proglacial lake sediments harbour distinct DNA viral communities that play important roles in shaping microbial community structure and mediating biogeochemical processes.

To address the knowledge gap in viral ecology, we conducted integrated metagenomic and metatranscriptomic analyses of sediments from Qiangyong proglacial lake, a representative proglacial lake on the southern TP. We aim to: (i) characterise the composition, function and activity of DNA viral communities in the proglacial lake sediments; (ii) explore the environmental factors that shape the viral communities and their interactions with hosts; (iii) evaluate the potential role of viral AMGs in mediating microbial metabolisms. Our results reveal an unexpectedly high diversity of DNA viruses—over 4000 putative species—and a broad repertoire of actively transcribed AMGs linked to carbon and nitrogen metabolism. These findings significantly expand our understanding of DNA viral diversity and their functional contributions in extreme cryosphere aquatic ecosystems undergoing rapid transformation.

## 2 Results and Discussion

### 2.1 QY Proglacial Lake Sediment Dominated by Novel and Lytic DNA Viruses

**FIGURE 1:**
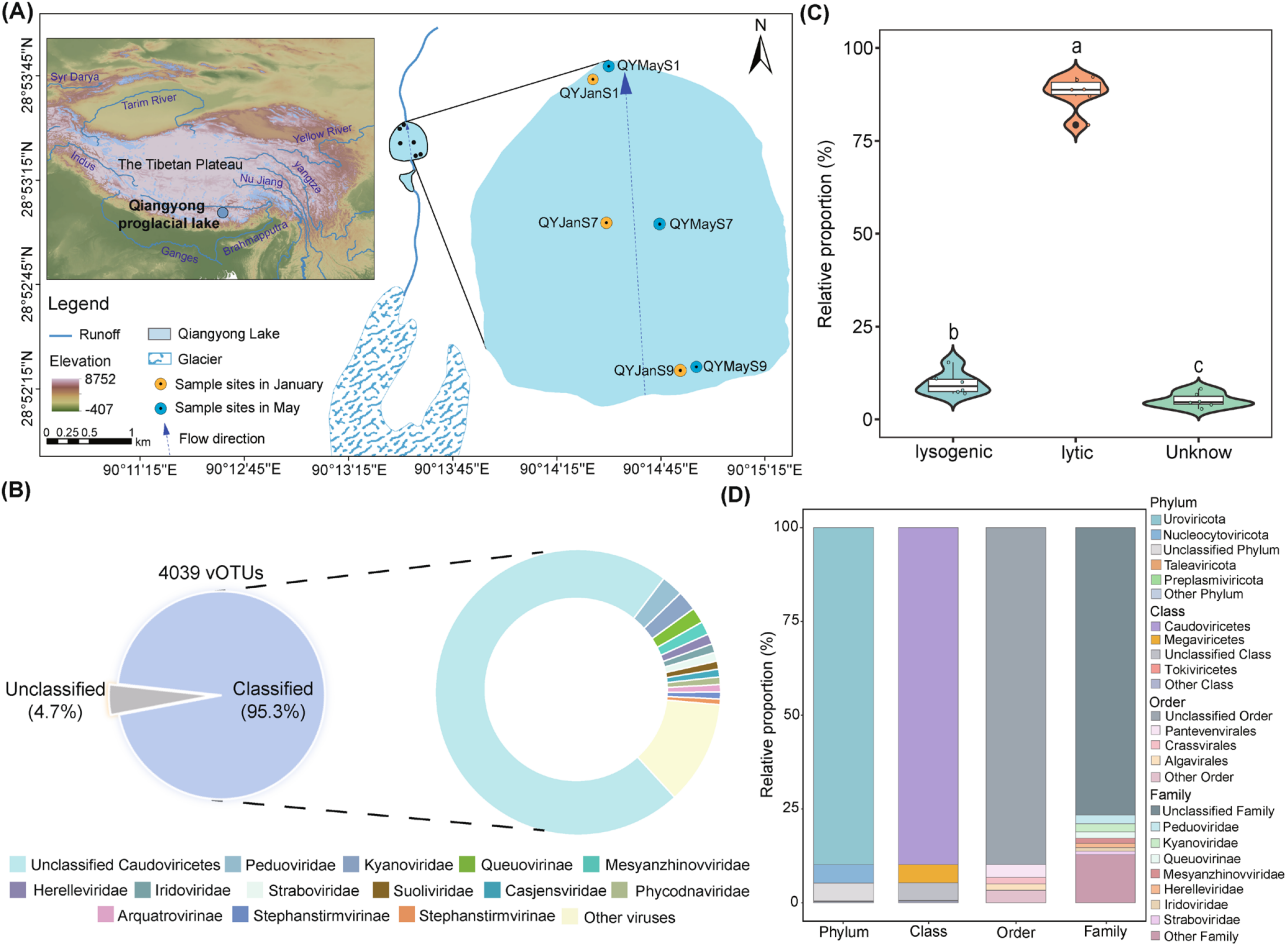
Overview of viruses in Qiangyong proglacial lake sediments. (A) Geographic distribution of collected Qiangyong proglacial lake sediment samples. (B) The proportions of classified and unclassified viruses (left) and the proportions of viral lineages at family level (right). (C) Relative proportions of lytic and lysogenic viruses in the Qiangyong proglacial lake sediments. The boxplots show the differences in the relative proportions of viruses with different lifestyles. Statistical significance was determined using a two‐tailed *t* test. (D) The proportion of viral community at the phylum, class, order and family level, respectively. The classification is based on viral operational taxonomic units (vOTUs), with the total vOTUs set as 100%. Taxonomic assignments were determined using a hierarchical integrated approach combining vConTACT2, BLASTn, geNomad, PhaGCN2 and VPF‐Class, with conflicts resolved via the Lowest Common Ancestor algorithm (see Section 4.4 for details).

To investigate DNA viral communities in proglacial lake sediments, we conducted metagenomic and metatranscriptomic sequencing on six sediment samples collected from Qiangyong (QY) proglacial lake on the southern TP during January and May. The sampling sites spanned from inlet to outlet, capturing the spatial variability across the QY proglacial lakes (Figure 1A). The metagenomic dataset was analysed using four viral identification methods, resulting in 5492 viral contigs (vContigs) with lengths ≥ 10 kb. These were dereplicated into 4039 viral operational taxonomic units (vOTUs) based on ≥ 85% alignment fraction and ≥ 95% average nucleotide identity (see Section 4.3 for details) (Figure 1B and Table S1). The genome size of vOTUs ranged from 10 to 321 kb, and 92.7% of them were between 10 and 60 kb (Table S1). Of these 4039 vOTUs, the majority (3385; 83.8%) were low‐quality genomes, followed by medium‐quality (385; 9.5%), complete (146; 3.6%), high‐quality (114; 2.8%) and quality‐not‐determined (9; 0.2%) genomes (Table S1). Life strategy predictions showed a remarkably higher proportion of lytic viruses (3319 vOTUs, 82.2%) than lysogenic viruses (430, 10.6%) (Figure 1C). This may be because lytic viruses can release large amounts of dissolved organic matter and nutrients by lysing the host, which is more conducive to maintaining the growth and metabolic activities of microbial communities in oligotrophic proglacial lake sediments (Danovaro et al. 2008; Weinbauer 2004), and thus be selectively enriched.

Taxonomic assignment of vOTUs showed that 3849 of 4039 vOTUs (95.3%) could be classified to known taxa using the Lowest Common Ancestor algorithm (see Section 4.4 for details) (Figure 1B). Among these, the QY proglacial lake sediment virus taxonomy covered 10 DNA virus phyla, mainly including *Uroviricota* (3630 vOTUs, 89.9%), *Nucleocytoviricota* (198 vOTUs, 4.9%), *Taleaviricota* (5 vOTUs, 0.1%) and *Preplasmiviricota* (4 vOTUs, 0.1%) (Figure 1D and Table S2). The majority of classified vOTUs (3849, 95.3%) could only be resolved at the class level (Figures 1D and S1 and Table S2). *Caudoviricetes* (3630 vOTUs, 89.9%) belonging to *Uroviricota* largely represented the taxonomic diversity of DNA viruses in QY proglacial lake sediments (Figures 1D and S1 and Table S2). Only 945 (23.4%) vOTUs can be resolved at the family level, suggesting that DNA viruses in proglacial lake sediments have high genetic diversity and harbour many novel DNA viruses that have not yet been detected. These vOTUs were distributed in 56 different families, mainly including *Peduoviridae* (95, 2.4%), *Kyanoviridae* (87, 2.2%), *Queuovirinae* (68, 1.7%) and *Mesyanzhinovviridae* (58, 1.4%) (Figure 1D). In addition, 376 vOTUs assigned to nucleocytoplasmic large DNA viruses (NCLDVs), primarily comprising the *Iridoviridae* (37) and *Phycodnaviridae* (27) (Table S2).

Viral transcriptional dynamics in QY proglacial lake sediments revealed that 15.0% (607) of vOTUs were active, with *Uroviricota* predominating (579, 95.4%), followed by *Nucleocytoviricota* (3, 0.5%) and *Preplasmiviricota* (1, 0.2%), alongside three unclassified vOTUs (Figure S2 and Table S2). This proportion of transcriptionally active viruses is comparable to other reported extreme oligotrophic environments, such as groundwater (15%) and deep‐sea brine pools (15%) (Gios et al. 2024; Minch et al. 2024), but much lower than nutrient‐rich environments, such as wastewater treatment plants (58%–76%) and rhizosphere soils (78%) (Muscatt et al. 2022; Shi et al. 2022), suggesting that resource availability in the ecological environment may have a significant influence on viral transcriptional activity. Specifically, in nutrient‐deprived ecosystems, the limited number of host cells available to viruses leads to reduced opportunities for replication and proliferation (Minch et al. 2024), with only a few viruses remaining active, while nutrient‐replete environments can promote host cell growth and proliferation, providing more opportunities for viruses to infect and enable them to replicate in large numbers (Muscatt et al. 2022), with a consequent increase in the number of active viruses.

In addition, transcriptional analyses at the family level revealed differences in activity hierarchies between viral taxa in the same ecosystem. For example, *Peduoviridae*, *Queuovirinae* and *Mesyanzhinovviridae* demonstrated both high abundance and transcriptional activity (Figures S1 and S2), suggesting that they may have more active biological functions and ecological roles. Conversely, *Kyanoviridae*, *Straboviridae* and *Vequintavirinae* exhibited low transcriptional activity relative to their abundance (Figures S1 and S2), suggesting that they may favor persistence over active replication in survival strategies. This activity‐abundance difference likely represents an evolutionary adaptation to the proglacial lake ecosystem, where metabolic dormancy enhances viral survivability during environmental stress while retaining rapid replicative potential under favorable conditions (Van Lint et al. 2013; Kercher and Mitchell 2002).

### 2.2 Virus–Host Linkages in QY Proglacial Lake Sediment

**FIGURE 2:**
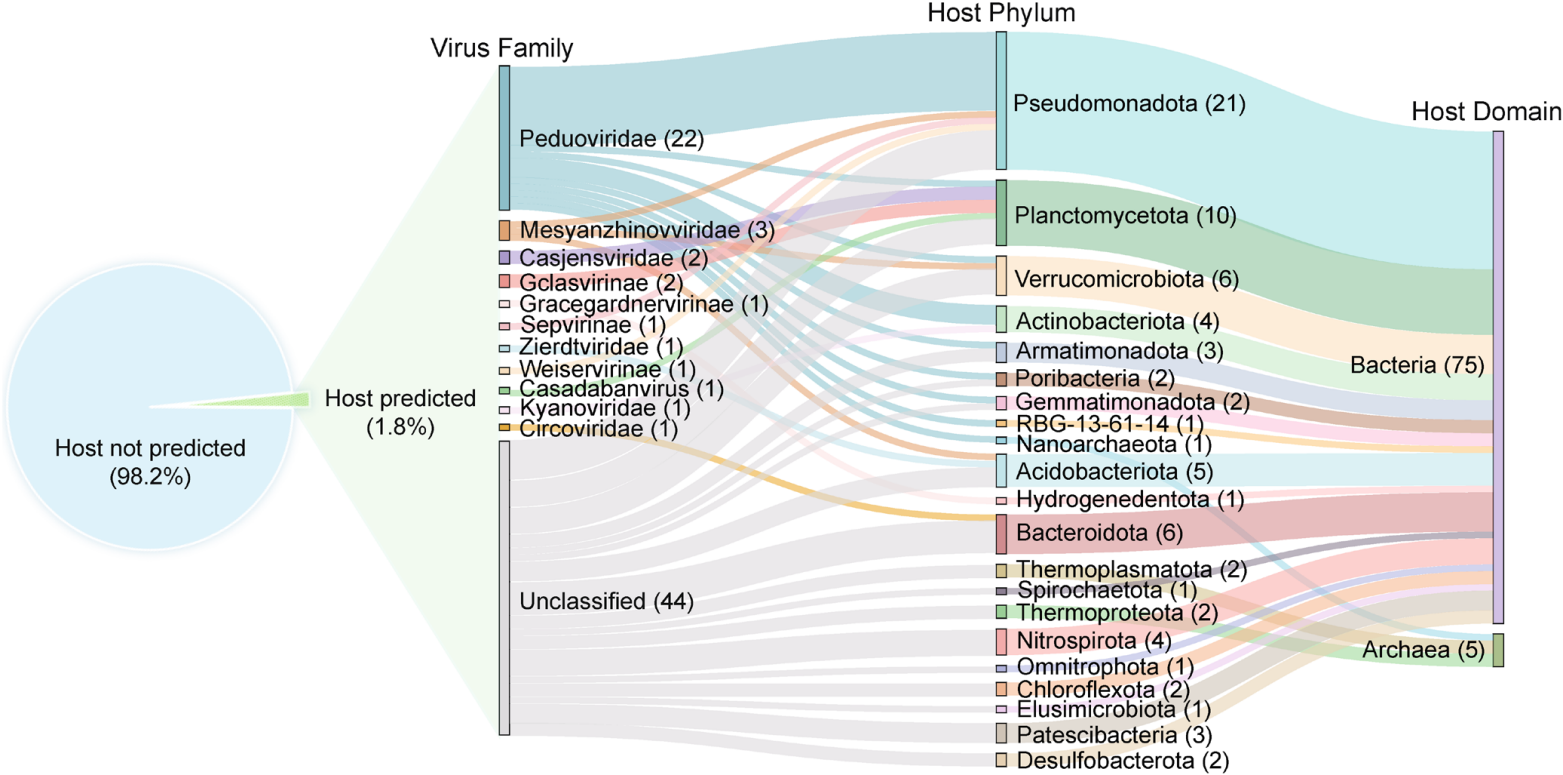
Predicted virus–host linkages. The pie chart shows the percentage of viral operational taxonomic units (vOTUs) with host prediction. The left, middle and right columns of the Sankey diagram show the viral family‐level taxonomy, predicted host phylum, and host domain, respectively.

Predicting the hosts of viruses is important for understanding virus–host interactions and potential co‐evolutionary mechanisms in QY proglacial lake sediment environments. Thus, we predicted both in situ and ex situ hosts to maximise the determination of host range using iPHoP (Roux et al. 2023). Ex situ hosts prediction based on default database linked 333 vOTUs (8.2%) to potential hosts, with the dominant bacterial hosts as Pseudomonadota, Bacteroidota and Actinomycetota, and the archaeal hosts as Thermoproteota, Asgardarchaeota and Nanoarchaeota (Table S3). Furthermore, in situ host prediction based on our own metagenome‐assembled genomes (MAGs) database resulted in 1.8% (71) of vOTUs being linked to 69 MAGs (Figure 2 and Table S4). Predicted proglacial lake sediment hosts comprised diverse prokaryotes, spanning 18 bacterial (64 MAGs) and three archaeal phyla (5 MAGs) (Figure 2 and Table S4). Of these, Pseudomonadota was the most common bacterial host phylum (26.3% of linkages), followed by Planctomycetota (12.5%), Verrucomicrobiota (7.5%) and Bacteroidota (7.5%). The predicted archaeal hosts included Thermoproteota (2.5%), Thermoplasmatota (2.5%) and Nanoarchaeota (1.3%). These results are consistent with the total relative abundance of these host phyla, all of which have a high proportion in the MAG dataset (Figure S3), suggesting viruses generally infect dominant hosts, likely due to higher encounter probabilities. However, we observed exceptions where highly abundant viruses infect less abundant hosts (Figure S4). For instance, the highly abundant vOTU QYJanS1.5_3655613 is linked to the low abundance phylum Spirochaetota.

In addition, the majority (87.0%) of these hosts are infected by a single virus. This result is consistent with the previous findings that most viruses have a narrow host range, which may be attributable to the specificity of phage recognition of host cell surface receptors (Paez‐Espino et al. 2016; Cheng et al. 2022; Chevallereau et al. 2022). Furthermore, some viruses encode defence systems that prevent the same host cell from being infected by other viruses (Hampton et al. 2020). However, nine host cells can be infected by multiple different viruses (average 2.2 ± 0.8 viruses per host) (Table S4). For example, QYJanS9_bin320, which belongs to Pseudomonadota, can be infected by three viruses of *Caudoviricetes* (QYJanS9.5_1423733, QYJanS9.5_1584918 and QYJanS9.5_184203). This phenomenon of multiple infections has been widely reported (Gios et al. 2024; Liu, Huang, et al. 2023; Roux et al. 2014; Díaz‐Muñoz 2017), and it may enhance genetic diversity and adaptive evolution of microbes via gene exchange between hosts and viruses (Stedman 2015). Furthermore, it can increase the selective pressure within the host cell, forcing microbes to develop more effective antiviral mechanisms or to resist viral attack by altering cellular metabolism (Labrie et al. 2010; Liu et al. 2019). Notably, these predictions of virus–host interactions are based on in silico tools. Although iPHoP employs a high confidence threshold and integrates multiple predictive evidence sources, predictions may still contain some false positives.

Antiviral defence systems were detected in analysed MAGs except QYJanS7_mtb2.113 and QYMayS1_mtb2.302, with restriction‐modification (RM) systems being most prevalent (75.4% of MAGs), followed by toxin‐antitoxin (TA, 31.8%) and CRISPR‐Cas systems (29.0%) (Table S5). The RM systems can methylate particular motifs of host DNA by a methyltransferase (MTase), while corresponding restriction endonuclease (REase) selectively cleaves differently methylated or unmethylated viral DNA (Tock and Dryden 2005). The toxin–antitoxin system employs an abortive infection (Abi) strategy that induces programmed cell death upon detection of viral infection to block phage propagation, while the CRISPR‐Cas system confers adaptive immunity via sequence‐specific cleavage of invading viral DNA using CRISPR RNA‐guided Cas nuclease (Lopatina et al. 2020). Although hosts have diverse defence strategies, persistent infections suggest that viruses have evolved complex mechanisms to escape host immunity. For example, four vOTUs can encode methyltransferases that protect their DNA from REase attack (Hampton et al. 2020) (Figure S5 and Table S6). Some phages have been shown to counteract the TA system by acquiring antitoxins or antitoxin mimics to neutralise their cognate toxins (Blower et al. 2012; Wan et al. 2016; Alawneh et al. 2016). We detected antitoxin homologues, including MazE, YefM, HigA, PrlF, MqsA and ParD, in four vOTUs (Table S6). However, we did not detect transcription of these coding genes, and the role of these proteins also deserves further investigation.

### 2.3 Environmental Drivers of Viral and Host Community Dynamics in Proglacial Lake Sediments

To understand the diversity of QY proglacial lake sediment viruses, the alpha diversity of viral communities was calculated. The results showed that viral Pielou's evenness ranged from 0.4 to 0.6, Shannon diversity index (H′) ranged from 4 to 7, and viral richness ranged from 700 to 3200 (Figure S6A–C). Notably, virus richness was significantly higher in proglacial lake sediments compared to other typical cryosphere habitats on the Tibetan Plateau, including snow (400–1700), ice (50–2000), meltwater (300–2000) and cryoconite (200–1200) (Liu, Jiao, et al. 2023). Spearman's correlation analysis of alpha diversity indexes with environmental factors revealed significant correlations only between the Shannon index and some environmental factors. Specifically, it was significantly positively correlated with pH and nitrate nitrogen (NO_3_^−^–N), but significantly negatively correlated with ammonium nitrogen (NH_4_^+^–N) (Figure S6D), revealing that these environmental factors have an important impact on the diversity of viral communities.

**FIGURE 3:**
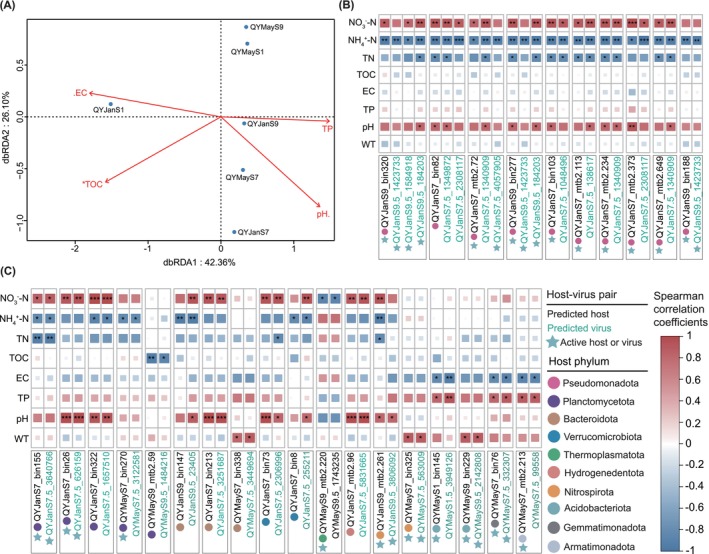
Impact of physicochemical parameters on viral community structure and host–virus relationships. (A) Distance‐based redundancy analysis (dbRDA) of bray‐Curtis dissimilarities between six proglacial lake sediments viral communities based on normalised coverage data. Vectors represent fitted environmental variables significantly correlated with dbRDA coordinates (permutation test, number of permutations = 999; . means *p* < 0.1, * means *p* < 0.05). (B) Correlation heatmaps of Pseudomonadota and their viruses (1–3 per host) with physicochemical properties (*p < 0.05, **p < 0.01 and ***p < 0.001). (C) Correlation heatmaps of remaining host lineages and their viruses (1–2 per host) with physicochemical properties (*p < 0.05, **p < 0.01 and ***p < 0.001). Only host–virus pairs with consistent correlations are shown. The size and colour of the squares indicate the Spearman correlation coefficient (*r*) between the individual indicators. Circles of different colours represent distinct host phyla. Star markers indicate hosts or viruses with transcriptional activity. Panels (B) and (C) share the same legend.

Furthermore, to investigate the environmental factors driving the composition of viral communities in proglacial lake sediments, we performed a distance‐based redundancy analysis (dbRDA) using relative abundance data of vOTUs. The dbRDA results revealed that environmental factors collectively explained a significant portion of the difference in viral community composition, with the first two dbRDA axes accounting for 42.4% and 26.1% of the total variance, respectively (Figure 3A). Among these physicochemical parameters, TOC had a significant effect (*p* < 0.05) on viral community structure in QY proglacial lake sediments, which is consistent with studies in other extreme environmental sediments such as hypersaline spring, Great Salt Lake, and subalpine peatland (Colangelo‐Lillis et al. 2016; Bhattarai et al. 2021; Xiong et al. 2025). In these oligotrophic environments, carbon limitation can modulate host microbial community structure through growth constraints, resulting in bottom‐up control of viral communities (Colangelo‐Lillis et al. 2016). Notably, TOC was also significantly correlated with viral transcriptional activity (Figure S7A), suggesting that it not only affects the structure of the viral community but may influence viral activities.

**FIGURE 4:**
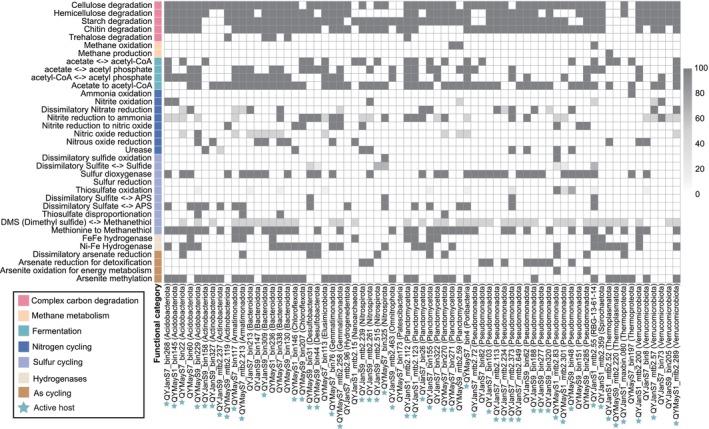
Functional potential of putative prokaryotic hosts. Heatmap showing the completeness of the metabolic pathways encoded by the putative host. KEGG KO of genes required for various metabolic and biosynthetic functions shown here were parsed using KEGG automatic annotation server and METABOLIC. Star markers indicate hosts or viruses with transcriptional activity.

Using Procrustes rotation and permutation analysis, we found that viral and host community members are closely linked in the QY proglacial lake sediments (Procrustes rotation correlation 0.980, *p* = 0.007) and that changes between these communities are coupled (Figure S7B), suggesting that host preferences also indirectly influence the composition of viral communities. Thus, we further assessed the effect of environmental factors on the predicted virus–host pairs. In total, 33 virus–host pairs (41.3%) were positively or negatively correlated with at least one of the eight measured environmental variables (Figures 3B,C and S8 and Tables S7 and S8). The environmental factor most commonly associated with virus–host pairs was NH_4_^+^–N content, with 20 pairs (25%) significantly negatively correlated with sediment ammonia concentration (Spearman's *ρ* < −0.80, *p* < 0.05) (Figures 3B,C and S8A and Table S8). NH_4_^+^ is an important nitrogen source for microorganisms (Morono et al. 2011). The increases of NH_4_^+^ concentration normally will lead to an increase of microbial abundance and their corresponding viruses (Wang et al. 2022) (similar to NO_3_^−^, see below). However, the opposite trend was observed here. The reason for this phenomenon remains unclear because our understanding of how NH_4_^+^ influences sediment viral community structure and virus–host interactions is still limited. Conversely, 17 virus–host pairs (21.3%) were significantly positively correlated with sediment nitrate concentrations (Figures 3B,C and S8B and Table S8). Members of the bacterial phyla linked to these viruses which include Pseudomonadota, Planctomycetota, Bacteroidota and Verrucomicrobiota were predicted to be involved in the nitrate reduction in our proglacial lake sediment samples (Figure 4) and other environments (Fan et al. 2024; Li et al. 2023). The presence of genes encoding nitrate reductase enables these microbes to thrive and reproduce in this nitrate‐limited environment (Kleinteich et al. 2022), which in turn influences the abundance of their linked viruses. Furthermore, approximately 36.3% of virus–host pairs were poorly correlated with physicochemical parameters, which may be due to differences between virus and host specific activities. For example, host defence mechanisms and viral life strategies (lytic or lysogenic) could regulate infection dynamics independently of environmental factors (Hampton et al. 2020; Jansson and Wu 2023). Such biological interactions in virus–host relationship models may partially blur physicochemical correlations in complex sediment matrices (Gios et al. 2024).

### 2.4 Viruses Are Related to Microbial Drivers of Biogeochemical Cycling in Proglacial Lake Sediments

To further reveal the influence of viruses on ecosystem function and biogeochemical cycling, we investigated metabolic potentials of virus hosts. As the main life forms in this system, sediment microorganisms orchestrate critical nutrient transformations—particularly of carbon (C), nitrogen (N) and sulphur (S)—that sustain ecosystem productivity (Kumar et al. 2022). Viruses can manipulate microbial metabolism and community structure via lysis and lysogeny strategies, thereby impacting biogeochemical cycles (Breitbart 2012). Based on the host–virus linkage, we found that most pathways regarding the nitrogen and sulphur cycle appear to be impacted by viral infections, including ammonia oxidation, dissimilatory nitrate reduction, denitrification, dissimilatory sulphate reduction and oxidation and thiosulfate oxidation (SOX) (Figure 4). Some of these influences occur through viral lysis. For instance, the ammonia‐oxidising archaea Thermoproteota (QYMayS7_bin197), encoding the rate‐limiting *amoABC* operon for nitrification initiation, exhibit CRISPR spacer matches to lytic *Caudoviricetes* viruses (QYMayS7.5_827513), suggesting viral pressure on a rate‐limiting nitrification step (Figure 4 and Table S4). Similarly, dissimilatory nitrate‐reducing bacteria Bacteroidota (QYMayS7_bin338), Planctomycetota (QYJanS7_bin26) and Pseudomonadota (QYJanS7_mtb2.373), which have complete pathways for reducing nitrate to ammonia, were also linked to lytic *Caudoviricetes* viruses (Figure 4 and Table S4). In addition, the detection of key genes revealed functional groups involved in denitrification (*nirK*, *nirS*, *norBC* and *nosZ*) and sulphur cycling (*sat*, *aprAB, dsr* and *sox*), with each of these MAGs linked to at least one lytic virus (24 vOTUs total) (Figure 4 and Table S4). Notably, one vOTU (QYJanS9.5_2190828) had an AMG encoding a nitrous oxide reductase (NosZ) that reduces nitrous oxide to nitrogen, but was not detected in its host (QYJanS9_bin158), suggesting that viruses may compensate for host nitrogen cycling through AMG‐mediated pathways. Although the host MAG exhibits high completeness (93.16%), we cannot entirely exclude the possibility of gene loss during assembly or binning. In addition, viruses (15 vOTUs) of heterotrophic bacteria capable of degrading complex polysaccharides have been identified (Table S6), and their metabolites, such as lactate and acetate, can fuel the cycling of nitrogen and sulphur (Baker et al. 2015), suggesting that viruses have the potential to influence multiple steps in the microbial food chain.

Hydrogenase‐mediated H_2_ cycling is critical for electron transfer and metabolism in anoxic sediments (Wrighton et al. 2014). We identified [NiFe]‐hydrogenase genes in 26 predicted hosts spanning 12 phyla (Figure 4) that have been shown to be associated with H_2_ oxidation during respiratory processes such as sulphate reduction and denitrification (Baker et al. 2015). While 12 hosts from fermentative taxa (*Thermoguttaceae*, *Aminicenantales* and *Bacteroidales*) encode [FeFe]‐hydrogenases that drive H_2_ production during organic matter degradation (Vignais and Billoud 2007). This microbial production of H_2_ is the central source of reducing power for *Methanomassiliicoccales* (QYJanS1_mtb2.52), the H_2_‐dependent methylotrophic methanogens, which lack the complete Wood‐Ljungdahl pathway for CO_2_ reduction and obligately depend on exogenous H_2_ to activate methyl‐coenzyme M reductase for methane production (Xie et al. 2024). *Methanomassiliicoccales* have been demonstrated to be one of the major taxa of methanogens in proglacial lake sediments (Huang et al. 2025), and our study discovered that it was also linked to a virus, which suggests that viruses may play an important role in regulating greenhouse gas emissions. Altogether, these results suggest that viruses have the potential to indirectly affect microbial carbon, nitrogen and sulphur cyclers in proglacial lake sediments.

### 2.5 Role of Auxiliary Metabolism Genes (AMGs)

**FIGURE 5:**
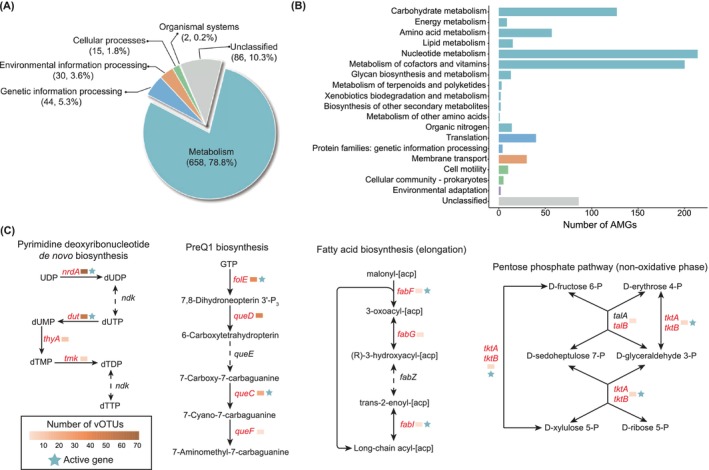
An overview of proglacial lake sediments virus‐encoded AMGs. (A) The percentage of viral AMGs involved in metabolism, genetic information processing, environmental information processing, cellular processes and organismal systems. (B) Distribution of 835 viral AMGs in 18 functional categories. (C) Four complete metabolic pathways targeted by AMGs which have previously been identified. AMGs are shown in red, and the activity of the AMG and the number of vOTUs are marked next to each AMG.

In addition to physically impacting microbial communities, viruses can carry AMGs that influence biogeochemical cycles by altering host metabolism (Breitbart et al. 2007). In total, 835 putative AMGs were identified in 420 vOTUs, which potentially participate in 59 biological pathways associated with metabolism (51), genetic (3) and environment (1) information processing, cellular processes (3) and organismal systems (1) (Figure 5A and Table S9). These pathways spanned 18 functional categories, with the metabolism of nucleotides (214, 25.6%), cofactors and vitamins (200, 24.0%), carbohydrates (127, 15.2%), amino acids (57, 6.8%), and translation (40, 4.8%) listed as the most common types (Figure 5B). Viruses with these AMGs were found in all six samples (Figure S9).

AMGs involved in purine, pyrimidine, folate, nicotinate and nicotinamide biosynthesis were overrepresented in proglacial lake sediment viral genomes compared to other metabolisms (Figure S10 and Table S9). These metabolic functions have been reported to be enriched in marine and supraglacial DNA viruses (Tian et al. 2024; Liu, Jiao, et al. 2023), which may indicate consistent selective pressures to acquire AMG from different habitats. Notably, four of the 59 metabolic pathways, including the pyrimidine deoxynucleotides biosynthesis, PreQ1 biosynthesis pathways, fatty acid biosynthesis (elongation) and pentose phosphate pathway (non‐oxidative phase), were considered complete or nearly complete (completeness ≥ 0.75), and most of the AMGs involved were detected to harbour transcriptional activity (Figure 5C), implying that they may be the key targeting pathways for viral AMGs in proglacial lake sediments. Some of these pathway steps are encoded by only one vOTU (e.g., *fabF, fabG* and *fabI* in the fatty acid biosynthesis pathway), whereas others are encoded by multiple vOTUs (e.g., *nrdA*, *dut*, *thyA* and *tmk* in pyrimidine deoxynucleotide synthesis), which may be related to their functional importance in the virus (Figure 5C). For example, nucleotides are essential for virus genome replication (Mahmoudabadi et al. 2017), and carrying nucleotide synthesis AMGs can modulate host metabolism and redirect it to provide resources for viral replication (Thompson et al. 2011). Viral AMGs encode enzymes involved in the synthesis of vitamins such as folate, nicotinate, nicotinamide and pantothenate, which are required for the efficient functioning of many biochemical processes such as DNA and amino acid synthesis (Liu, Jiao, et al. 2023; He et al. 2024). In addition, AMGs related to carbon (e.g., fructose/mannose, starch/sucrose, galactose, butanoate and pyruvate), nitrogen (nitrous oxide reductase *nosZ*, K00376) and phosphorus (phosphate transport system substrate‐binding protein *pstS*, K02040; and phosphate starvation‐inducible protein *phoH*, K06217) metabolism were identified, which help the host overcome nutrient and energy limitations in proglacial lake sediments while contributing to biogeochemical cycling.

**FIGURE 6:**
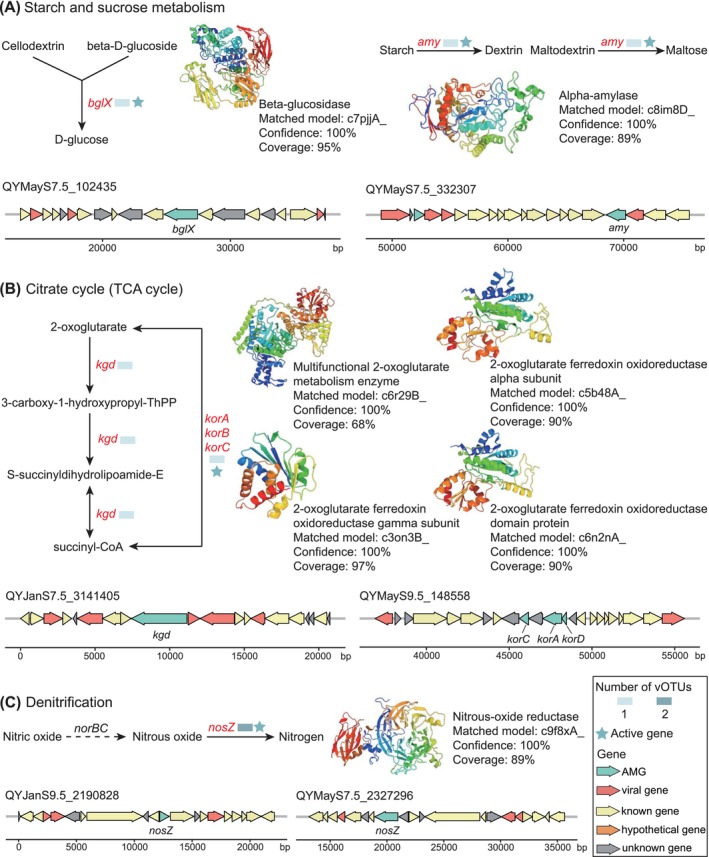
The metabolic pathways associated with novel AMGs. (A) Metabolic pathways with *bglX* and *amy* genes, the reference protein models for the BglX and AMY, and genome architecture of vOTU encoding *bglX* and *amy* genes. (B) The potential contribution of AMGs to the tricarboxylic acid (TCA), reference protein models for KGD, KorA, KorB and KorC, and the genome architecture for the vOTUs encoding TCA genes. (C) The potential contribution of AMGs to denitrification, the reference protein model for NosZ, and the genome architecture for the vOTUs encoding the *nosZ* gene. QYJanS9.5_2190828 and QYMayS7.5_2327296 share the same NosZ protein structure model. AMGs are shown in red, and the activity of the AMG and the number of vOTUs are marked next to each AMG. Panels (A), (B) and (C) share the same legend.

Beyond exploring the metabolic pathways that AMGs were involved in, we also investigated whether viruses in the QY proglacial lake sediment encoded AMGs that were not reported in the AMG reference dataset by Tian et al. (2024). We identified 63 such AMGs (29 active) out of 199 different AMGs, which are primarily associated with hydrocarbon metabolism (Table S9). These AMGs encode beta‐glucosidase (*bglX*, K05349) and alpha‐amylase (*amy*, K01176) in starch and sucrose metabolism, and multifunctional 2‐oxoglutarate metabolism enzyme (*kgd*, K01616) and 2‐oxoglutarate ferredoxin oxidoreductase (*korA*, K00174; *korC*, K00177; and *korD*, K00176) in the tricarboxylic acid (TCA) cycle. Protein structure predictions supported these functional annotations with 100% confidence for each of these AMGs, with the *bglX*, *amy* and *kor* genes being transcriptionally active (Figure 6). During carbohydrate metabolism, *bglX* and *amy* would catalyse the hydrolysis of polysaccharides (e.g., cellulose, starch and maltose) (Figure 6A), while *kor* would compensate for host TCA cycle by converting 2‐oxoglutarate to succinyl‐CoA (Figure 6B). Furthermore, we found the first viral AMGs‐encoded NosZ in QYJanS9.5_2190828 and QYMayS7.5_2327296, structurally and actively confirmed to catalyse nitrous oxide to nitrogen reduction (Figure 6C), a discovery expanding viral roles in nitrogen cycling (Wang et al. 2022; Gazitúa et al. 2021). Collectively, these AMGs may optimise host competitiveness by expanding substrate utilisation and enhancing core metabolism together (Emerson et al. 2018), thereby creating metabolic niches that maximise viral progeny production.

**FIGURE 7:**
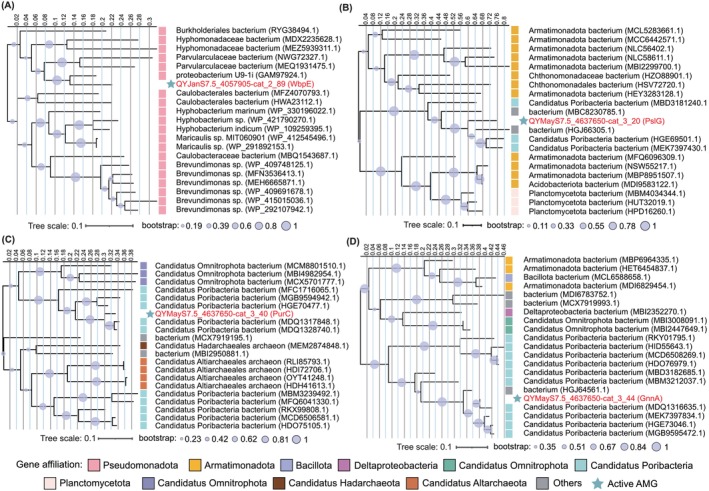
The AMGs that are without homologs to the putative host. (A) The phylogenetic tree of WbpE. (B) The phylogenetic tree of PslG. (C) The phylogenetic tree of PurC. (D) The phylogenetic tree of GnnA.

We found some functional redundancy between AMGs and host metabolism when comparing the predicted AMG sequences to their host genomes. Specifically, 47 AMGs shared homology with host genes (> 40% amino acid identity over 60% length of viral query sequence), of which 27 had 100% identity (Table S10). Viruses may duplicate host functions to overcome metabolic bottlenecks in resource biosynthesis during viral reproduction in host cells (Gios et al. 2024). For example, six ribosomal proteins (RPS6e, RPL10e, RPL11, RPL21e, RPS3Ae and RPS28e) were detected in QYJanS1.5_1362031 that were homologous to genes in its putative host QYJanS1_mtb2.15 (100% protein sequence identity) (Table S10). After infection, viruses can use their own ribosomal proteins to usurp endogenous translation pathways, selectively inhibit host protein synthesis, and increase viral protein biosynthesis, thereby optimising their reproductive efficiency (Mizuno et al. 2019). In contrast, five active AMGs, including UDP‐2‐acetamido‐2‐deoxy‐ribo‐hexuluronate aminotransferase (*wbpE*, K13017), polysaccharide biosynthesis protein (*pslG*, K21000), phosphoribosylaminoimidazole‐succinocarboxamide synthase (*purC*, K01923), UDP‐N‐acetylglucosamine 3‐dehydrogenase (*gnnA*, K18855) and nitrous oxide reductases (*nosZ*, K00376), showed no homology to host genes, but the phylogenetic tree shows that the origin of these AMGs is consistent with their host phylum (Figures 7 and S11A and Table S10). This may be due to the fact that host genes were lost in evolution, but the virus retained these genes and maintained their functions through horizontal gene transfer (HGT). Notably, AMGs encoding isocitrate lyase (*aceA*, K01637) and ribonucleoside‐diphosphate reductase alpha chain (*nrdA*, K00525) not only lacked host homologues but also originated from other phyla (Figure S11B), implying that virus‐mediated cross‐phyla HGT introduces new functions to the host metabolic repertoire and facilitates ecological niche adaptation.

Of the 835 AMGs, the transcription of 107 (12.8%) were detected in the metatranscriptomes (Figure S12A and Table S11), further confirming their potential impact on the proglacial lake sediment ecosystem. We found that AMGs related to carbohydrate metabolism (35, 32.7%), membrane transport (15, 14.0%) and amino acid metabolism (13, 12.1%) were the top three in the metatranscriptome (Figure S12B and Table S11), which was different from the distribution of the AMGs repertoire (Figure 5). This transcriptional bias may reflect an adaptive strategy of viruses in oligotrophic environments, where viruses enhance the metabolic competitiveness of their hosts by preferentially expressing AMGs associated with energy acquisition and nutrient transport, thereby sustaining their own proliferation under energy‐limited conditions. Alternatively, it could be attributed to certain AMGs being expressed only under specific conditions or inadequate sequencing depth of the metatranscriptome. Of the transcribed AMGs, transketolase (*tktA*, K00615) and DNA (cytosine‐5)‐methyltransferase (*dcm*, K00558) were the most common (Table S11). Virus‐encoded TktA can help host cells catalyse ketol group transfer reactions in the pentose phosphate pathway and generate important intermediates such as ribose‐5‐phosphate and erythrose‐4‐phosphate, thereby enhancing nucleotide precursor synthesis and NADPH generation (Sprenger 1995). DNA methyltransferases aid in viral capsid stabilisation, help circumvent organic nitrogen limitation through methionine degradation, and provide immunity to the RM system when the virus infects the host (Robinson et al. 2024; Murphy et al. 2013). Overall, our results suggest that proglacial lake sediment viruses encode diverse AMGs that are capable of hijacking and enhancing host metabolic networks, thereby covertly directing biogeochemical cycling processes in the cryosphere ecosystem.

*Limitations*: The dataset contains a substantial proportion of viral genomes with incomplete or fragmented sequences. Although all viral sequences were identified under stringent prediction thresholds and validated by CheckV to contain at least one viral hallmark gene (Luo et al. 2022), the limited completeness of many vContigs may affect the accuracy of taxonomic classification and functional annotation. In particular, the high genetic novelty of viruses in proglacial lake sediments—coupled with the scarcity of closely related reference genomes—likely contributed to the low rate of family‐level classification. However, we cannot rule out that assembly fragmentation also limited taxonomic resolution in some cases. Furthermore, the quality and completeness of both vOTUs and MAGs may influence the sensitivity of host prediction, thereby affecting the interpretation of virus–host interaction networks. Although the use of both in situ and *ex situ* host prediction strategies aimed to maximise coverage, some associations remain tentative. Despite these limitations, the inclusion of partial and fragmented genomes still offers valuable insights into the composition and ecological activity of viral communities in proglacial lake sediments. Future studies leveraging long‐read sequencing and complete genome recovery will help clarify these associations and improve the resolution of viral taxonomy and host linkages (Roux et al. 2019).

## 3 Conclusion

In summary, this study provides a comprehensive analysis of DNA virus communities in QY proglacial lake sediments, elucidating their taxonomic composition, host interactions and potential functions in mediating sediments biogeochemical cycling. We found that viruses in proglacial lake sediments are not only numerous and diverse, but also harbour substantial unexplored viral lineages. Host predictions revealed that viruses can influence biogeochemical cycles by infecting microbial taxa responsible for key nutrient transformations. The discovery of antiviral and counter‐defences systems underscores the ongoing evolutionary arms race between microbes and viruses. In addition, AMGs were identified and suggested that viruses influence carbon, nitrogen and phosphorus cycling, as well as nucleotide and cofactors/vitamins metabolism, which indirectly participate in biogeochemical cycles. These findings advance our understanding of the virus diversity in proglacial lake sediments and its impact on host metabolism and biogeochemical cycling.

## 4 Methods

### 4.1 Sample Collection and Physicochemical Measurement

Qiangyong proglacial lake (28.892° N, 90.226° E; 4870 m above sea level) is situated on the southern Tibetan Plateau between the Himalayan Mountains and the Yarlung Tsangpo River, with a surface area of ∼0.8 km^2^ and an average water depth of ∼17 m (Zhang, Xu, et al. 2023). We collected six surface sediment samples (~15 cm) by grapple at three sites spanning from the inlet to the outlet of Qiangyong proglacial lake in January and May 2023, respectively, and then transferred into 500 mL sterile Whirl‐Pak bags (Nasco, Fort Atkinson, USA). After sample collection, sediments were immediately transported to the laboratory on dry ice and stored at −80°C prior to downstream procedures.

Sediment physicochemical properties were characterised as previously described (Liu, Vick‐Majors, et al. 2017; Liu et al. 2013). The pH and electrical conductivity (EC) were measured by the potentiometric method after mixing the sediment with distilled water at a ratio of 1:5 (g/g). The total nitrogen (TN) content was determined using the Kjeldahl method (Wang and Oien 1986). Ammonium nitrogen (NH_4_^+^–N) and nitrate–nitrogen (NO_3_^−^–N) were extracted from fresh sediment with 2 M KCl (sediment to solution ratio = 1:5) using the Smartchem200 Discrete Auto Analyser (Skalar, Breda and Netherlands). Total phosphorus (TP) was determined using the Smartchem200 Discrete Auto Analyser. Total organic carbon (TOC) was determined on a milled sample by combustion at 990°C using the TOC Analyser (Shimadzu Corp, Japan).

### 4.2 DNA Extraction, Sequencing, Assembly and Binning

The total DNA of each sample was extracted from 0.5 g sediment using the DNeasy PowerSoil Kit for Soil (QIAGEN, Hilden, Germany) following the manufacturer's instructions. The concentration of extracted DNA was measured using a Qubit 4.0 Fluorometer (Thermo Fisher Scientific). The sequencing libraries of DNA were constructed using the KAPA Hyper Prep Kit (Kapa Biosystems, Wilmington, USA) according to the manufacturer's instructions and sequenced using the Illumina NovaSeq 6000 platform with 2 × 150 bp paired‐end chemistry at Guangdong Magigene Biotechnology Co. Ltd. (Guangzhou, China).

Trimmomatic v0.39 (Bolger et al. 2014) with default parameters was used to remove adapters and filter low‐quality raw reads. Then, clean reads of each sample were individually assembled using MEGAHIT v1.2.9 (parameters: –min‐contig‐len 500 –k‐min 21 –k‐max 141) (Li et al. 2016). The assembled contigs were used to bin the metagenome assembled genomes (MAGs) using MaxBin2 v2.2.7 (Wu et al. 2016), MetaBAT2 v2.0 (Kang et al. 2019), VAMB v3.0.2 (Nissen et al. 2021) and BASALT v1.1.0 (Qiu et al. 2024). The original MAGs were further quality‐checked with CheckM v1.0.12 (Parks et al. 2015), and low‐quality bins (completeness < 50% and contamination > 10%) were filtered out. Subsequently, the obtained MAGs were dereplicated using dRep v3.3.0 (Olm et al. 2017) with an average nucleotide identity (ANI) cutoff value of 99%. The recovered MAGs were taxonomically assigned using GTDB‐Tk (v2.4.0, reference data version r220) (Chaumeil et al. 2022). The metabolic pathways of MAGs were annotated using KAAS (KEGG Automatic Annotation Server: http://www.genome.jp/kegg/kaas/) (Moriya et al. 2007), METABOLIC v4.0 (Zhou et al. 2022) and DRAM v1.4.6 (Shaffer et al. 2020) with default parameters.

### 4.3 Identification, Quality Checking and Clustering of Viral Contigs

Viral contigs (vContigs, ≥ 10 kb) were first identified from metagenome assemblies using geNomad v1.8.0 (Camargo et al. 2024), Virsorter2 v2.2.1 (Guo et al. 2021), DeepVirFinder v1.1 (Ren et al. 2020) and VIBRANT v1.2.1 (Kieft et al. 2020) with default parameters. Then, the vContigs were screened and retained according to the following criteria: (i) score ≥ 0.8 for geNomad, (ii) score > 0.9 for VirSorter2, (iii) score ≥ 0.9 and *p* < 0.05 for DeepVirfinder, (iv) all viral contigs from VIBRANT. Putative vContigs from the four pipelines were merged, and the quality was checked and trimmed using CheckV v1.0.1 (Nayfach et al. 2021). The obtained vContigs were clustered at 95% ANI and 85% alignment fraction of the shortest vContigs using the CheckV script (https://bitbucket.org/berkeleylab/checkv/src/master/) to generate non‐redundant vOTUs.

### 4.4 Taxonomic Classification and Lifestyle Prediction of vOTUs

The obtained vOTUs were taxonomically classified using geNomad v1.8.0 (Camargo et al. 2024), vConTACT2 v0.11.3 (Jang et al. 2019), PhaGCN2 v2.0 (Jiang et al. 2023), VPF‐Class (Pons et al. 2021) and viral genome homologue search method based on BLASTn comparison of the IMG/VR V4 database (Camargo et al. 2023). Briefly, geNomad with the default parameter was used to classify vOTU by taxonomic rank of annotated proteins. For vConTACT2, prodigal v2.6.3 (−p meta) (Hyatt et al. 2010) was first used to predict open reading frames (ORFs). The resulting protein sequence was then used for vConTACT2 and the NCBI ‘ProkaryoticViralRefSeq201‐Merged’ database was selected as the reference database. A vOTU was considered to belong to a viral family when more than 50% of its proteins were assigned to the viral family with a bit score ≥ 50 (Jian et al. 2021). PhaGCN2 classified vOTUs at the family‐level using recommended cutoff scores > 0.5 (Jiang et al. 2023). VPF‐Class applied conservative membership ratio = 0.5 and confidence score = 0.75 for family‐ and genus‐level taxonomic assignment (Pons et al. 2021). The BLASTn‐based approach assigned to vOTU the top best hit virus taxonomic classification with at least 90% identity and 75% alignment coverage to the IMG/VR V4 database (Jarett et al. 2020). The final taxonomic assignment for each vOTU was determined through hierarchical integration of results from the five pipelines, prioritised in the following order: vConTACT2, BLASTn, geNomad, PhaGCN2 and VPF‐Class. If conflicts existed at a lower taxonomic level (e.g., family level) for different pipelines but there was a common higher level of taxonomy (e.g., order level), then the higher taxonomic level was assigned. In addition, nucleocytoplasmic large DNA viruses (NCLDVs) were identified using ViralRecall v2.1 (Aylward and Moniruzzaman 2021) with default parameters, where vOTUs with scores > 0 were classified as potential NCLDVs.

The lifestyle of vOTUs was predicted using three methods. (i) CheckV v1.0.1 predicts viral genome lysogenic lifestyle by detecting provirus boundaries. (ii) The vOTUs with proteins annotated (see below, DRAMv annotation of viral proteins) as integrases, invertase, serine recombinases, transposase, CI/Cro repressor and *parA/B* are considered proviruses (Jian et al. 2021). (iii) BACPHLIP v0.9.6 (minimum score ≥ 0.9, https://github.com/adamhockenberry/bacphlip) and VIBRANT v1.2.1 with default parameters were used to infer the lifestyle of the viral genome. The combined result of more than two software was used as the final prediction result. Conflicting results between two software were resolved through sequential priority assignment to CheckV, gene annotation, VIBRANT and BACPHLIP outputs.

### 4.5 Virus–Host Predictions and Identification of Anti‐Viral Defence Systems

To depict virus–host linkages, we predicted potential hosts for vOTU using iPHoP v.1.3.3 with the confidence score cutoff equal to 90, which combines phage‐based methods (RaFAH) and host‐based methods (BLAST, CRISPR, VirHostMatcher, WIsH, PHP) (Roux et al. 2023). To enhance the accuracy and reliability of host prediction, the MAGs recovered in this study were integrated into the host database using the ‘add_to_db’ module in iPHoP.

Anti‐phage defence systems were identified among putative host MAGs using DefenseFinder v2.0.0 (parameters: –db‐type gembase) (Tesson et al. 2022; Néron et al. 2023), updated with all known anti‐phage systems in February 2025 (defence‐finder‐models v2.0.2).

### 4.6 AMGs Identification

Functional annotations of gene and predicted AMGs for all vOTUs were made using the DRAM‐v pipeline (Shaffer et al. 2020). Briefly, all vOTUs were reanalyzed using VirSorter2 (parameters: ‐prep‐for‐dramv‐min‐length 5000‐min‐score 0‐include‐groups dsDNAphage, ssDNA, NCLDV, RNA, lavidaviridae) to generate an ‘affi‐contigs.tab’ file, which was subsequently annotated using DRAM‐v with the default database. The ‘‐min‐score 0’ parameter was employed to ensure maximal sensitivity, as some vOTUs derived from geNomad, DeepVirFinder and VIBRANT were not classified as viral contigs by VirSorter2 when using the ‘‐min‐score 0.5’ parameter, as previously described (Liu, Jiao, et al. 2023). Predicted AMGs were filtered based on the following criteria: auxiliary_score ≤ 3 and amg_flag assigned to M or M with E and/or K. In addition, the flanking genes of the putative AMG were manually inspected to ensure the presence of viral marker genes or viral‐like genes. To provide insight into the potential functional relevance of AMG to the host, we first compared vOTU sequences to the host genome using BLASTn (parameters: ‐perc_identity 100 ‐qcov_hsp_perc 100 ‐evalue 1e−3) to determine that the host genome was unintegrated or uncontaminated with viral sequences. Subsequently, the AMG was compared to the host genome using BLASTp to assess the relatedness of the identified AMG to homologous genes found in the host (Gios et al. 2024).

To determine whether the identified AMGs had been previously reported in existing AMG datasets, we used a protein clustering approach. Specifically, we used MMSEQS2 (Steinegger and Söding 2017) to cluster the previously published 25,281 AMG amino acid sequences by Tian et al. (2024). with the 835 AMG sequences identified in our study with the following parameters ‐min‐seq‐id 0.3 ‐c 0.6 ‐s 7.5. AMGs that did not cluster with any sequences in this reference set were regarded as not having been reported in that particular study. To confirm the functions of these AMGs, we applied the Phyre2 pipeline (http://www.sbg.bio.ic.ac.uk/phyre2) for structural analysis (Kelley et al. 2015).

### 4.7 Phylogenetic Analysis of AMGs

To investigate the probable evolutionary origin of the AMGs, homologue sequences of AMGs were recruited from the NCBI nr database (Release April 2025), and the top 20 hits with a bit score > 50 were retained as reference sequences for the AMGs. The AMGs and reference sequences were aligned using MUSCLE5 (Edgar 2022), and the alignment of sequences was filtered using trimAl v1.5.0 (−gappyout parameter) (Capella‐Gutiérrez et al. 2009) to remove sequences with > 70% gaps. The final multiple sequence alignment was used for maximum‐likelihood tree construction using MEGA software with Poisson model (Tamura et al. 2013; Hall 2013). The tree topology was evaluated using the bootstrap analysis based on 1000 resampling replicates. Phylogenetic trees were visualised using the iTOL online server (Letunic and Bork 2007). We regard the sources of sequences that cluster closely with AMG in the phylogenetic tree as potential sources of AMG.

### 4.8 Calculation of Abundance of MAGs, vOTUs and AMGs in Metagenomes

The relative abundance of MAGs and vOTUs was all calculated using Bowtie2 v2.4.5 (Langmead and Salzberg 2012) and coverM v0.6.1 (https://github.com/wwood/CoverM). Briefly, clean reads from each trimmed metagenome were mapped to the dereplicated MAGs and vOTUs nucleotide sequences using Bowtie2 with the ‘‐sensitive’ parameter, and the resulting bam file was sorted using Samtools v1.15.1 (Li et al. 2009). Transcripts per million (TPM) were generated based on the sorted bam files using coverM with ‘‐min‐covered‐fraction 10 ‐m tpm’ parameters. The TPM values of MAGs and vOTUs were estimated using the ‘genome’ and ‘contig’ commands, respectively. We used the abundance of vOTU to represent the abundance of their AMGs to avoid the potential impact of host gene‐derived reads on the estimation of the abundance of virus‐encoded AMGs (Jian et al. 2021).

### 4.9 Total RNA Extraction, Quality Checking and Sequencing

Total RNA was extracted from six samples with the RNAeasy PowerSoil Total RNA Kit (QIAGEN, Hilden, Germany) according to the manufacturer's instructions. Residual DNA removal and RNA cleaning were carried out using DNase I and the RNA Clean&Concentrator‐5 (Zymo, Irvine, USA), respectively. The quantity and quality of total RNA were measured using the RNA HS Assay Kit (Thermo Fisher Scientific, Waltham, USA) and Bioanalyzer 2100 (Agilent, Santa Clara, USA). Ribosomal RNA (rRNA) was removed with Ribo‐off rRNA Depletion Kit V2 kits (Vazyme, Nanjing, China). The library was prepared with VAHTS Universal V6 RNA‐seq Library Prep Kit (Vazyme, Nanjing, China) for Illumina following the manufacturer's recommendations. Sequencing was carried out using the Illumina NovaSeq 6000 platform with pair‐end 2 × 150 mode at Guangdong Magigene Biotechnology Co. Ltd. (Guangzhou, China).

### 4.10 Transcript Abundance of MAGs, vOTUs and AMGs Analysis

Metatranscriptomic raw reads were trimmed using Trimmomatic (Bolger et al. 2014) with default parameters to cut adapter sequences. Prokaryotic and eukaryotic rRNA reads were subsequently removed from total RNA with SortMeRNA v4.3.6 (Kopylova et al. 2012) with default parameters. Strand orientation was assessed for each sample using the infer_experiment.py script from RSeQC v5.0.4 (Wang et al. 2012). The mRNA reads for each sample were mapped to the nucleotide sequences of MAGs and vOTUs using Bowtie2 (Langmead and Salzberg 2012) with the sensitive mode. The mapped reads were further filtered using Samtools with ‐q 30 option (Li et al. 2009) to keep only high‐quality mappings. The TPM values of MAGs and vOTUs were calculated using CoverM with the ‐m tpm option. For TPM of AMGs, transcriptomic reads for vOTUs were first mapped to nucleotide sequences of AMGs using Bowtie2, and then calculated using Samtools and CoverM with the parameters as described above. MAGs, vOTUs and viral AMGs were considered active when the TPM value was greater than zero (Zhu et al. 2025).

### 4.11 Statistical Analyses and Plotting

All statistical analyses in this study were performed using R v4.1.4. Alpha diversity (richness, Shannon and evenness index) of viral communities was calculated using the ‘vegan’ package v2.5–7. The virus community relative abundance data and chemical characterisation were used to perform a PERMANOVA analysis in R with ‘vegan’, and then physicochemical parameters with *p* > 0.1 were selected for a distance‐based redundancy analysis (dbRDA) of bray‐Curtis dissimilarities to reveal potential environmental drivers of the virus community. In addition, Procrustes rotations and permutations were performed using the PROTEST function in ‘vegan’ to search for significant coupling between viral and their host communities. Spearman correlation analyses between normalised coverage of each genome (viral or prokaryotic) and each physicochemical parameter were performed using the R base ‘Hmisc’ package to reveal relationships between viral abundance, their hosts and environmental parameters. The manuscript figures were mainly generated using the R packages ‘ggplot2’, ‘ggpubr’, ‘dplyr’, ‘gggene’ and ‘pheatmap’. The figure format was adjusted using Adobe Illustrator if needed.

## Author Contributions

**Yang Zhao:** data curation (lead), formal analysis (lead), software (lead), writing – original draft (lead), writing – review and editing (lead). **Meiling Feng:** data curation (equal), formal analysis (equal), writing – review and editing (equal). **Hongfei Chi:** validation (supporting) and writing – review (supporting). **Keshao Liu:** conceptualization (equal), investigation (supporting), and writing – review (supporting). **Rong Wen:** validation (supporting) and writing – review (supporting). **Weizhen Zhang:** validation (supporting) and writing – review (supporting). **Pengfei Liu:** funding acquisition (lead), project administration (lead), resources (lead), conceptualization (equal), writing – review and editing (equal).

## Funding

This work was supported by the National Natural Science Foundation of China for Excellent Young Scientists Fund Program (42222105), the National Natural Science Foundation of China General Program (42171144), the Young Scientists Fund of the National Natural Science Foundation of China (42201056), the Key Research and Development Program of Gansu Province (24YFFA006), the Key Research and Development Plan of Tibet Autonomous Region (XZ202301ZY0008G), and the Global Ocean Negative Carbon Emissions (ONCE) Program.

## Conflicts of Interest

The authors declare no conflicts of interest.

## Supporting information


**Table S1:** Quality of the genomes of QY proglacial lake sediment virus populations (vOTUs).
**Table S2:** Taxonomic classification of QY proglacial lake sediment viral populations (vOTUs).
**Table S3:** Predicted ex situ hosts of vOTUs in the QY proglacial lake sediment environments.
**Table S4:** Predicted in situ hosts of vOTUs in the QY proglacial lake sediment environments.
**Table S5:** Defences systems found in the host using DefenseFinder.
**Table S6:** DRAM annotation results for 71 vOTUs associated with the host.
**Table S7:** Summary of Spearman correlation results between abundances of virus/host pairs and sediment physicochemistries (significant: *p* < 0.05).
**Table S8:** Full list of Spearman correlation results between abundances of virus/host pairs and sediment physicochemistries.
**Table S9:** Auxiliary metabolic genes (AMGs) detected in QY proglacial lake sediment viral population genomes.
**Table S10:** Functional redundancy between AMGs and putative host genes based on blastp results.
**Table S11:** Expressed AMGs detected in QY proglacial lake sediment virus population genomes.

**Figure S1:** The relative abundance (TPM) of the virus community. (A) Relative abundance of vOTUs across samples at class level. (B) Relative abundance of vOTUs across samples at family level.
**Figure S2:** The transcripts abundance of the virus community. (A) Transcript abundance of viral community at the phylum, class, order and family level, respectively. (B) Transcript abundance of vOTUs across samples at class level. (C) Transcript abundance of vOTUs across samples at family level.
**Figure S3:** Rank abundance plot showing the total relative abundance of 1336 prokaryotic MAGs across six Qiangyong proglacial lake sediment samples. Abundances were summed across sites and by phyla.
**Figure S4:** Distribution of relative abundance of host phyla associated with viruses.
**Figure S5:** The genomic architecture of vOTUs with selected potential anti‐defence system. Others could be found in Table S6.
**Figure S6:** The α diversity and influencing factors of the viral community in Qiangyong proglacial lake sediments based on metagenomic. (A) The richness index of virus communities in six Qiangyong proglacial lake sediment samples. (B) The Shannon index of virus communities in six Qiangyong proglacial lake sediment samples. (C) The Evenness index of virus communities in six Qiangyong proglacial lake sediment samples. (D) Key environmental factors significantly associated with viral α‐diversity (* means *p* < 0.05 and ** means *p* < 0.01).
**Figure S7:** Impact of biotic and abiotic factors on viral communities. (A) Distance‐based redundancy analysis (dbRDA) of bray‐Curtis dissimilarities between six proglacial lake sediments viral communities based on transcript abundance and physicochemical parameters. Vectors represent fitted environmental variables significantly correlated with dbRDA coordinates (permutation test, number of permutations = 999; . means ***p < 0.1, * means *p* < 0.05). (B) Procrustes rotation and substitution based on metagenomic abundance visualizes the coupling between viral and bacterial communities. Using Bray‐Curtis distances, bacterial and viral ordinations are rescaled and connected by a line.
**Figure S8:** Relationships between environmental factors and virus–host pairs. (A) Scatter plot depicting the correlation between NH_4_^+^–N and virus–host pairs. (B) Scatter plot depicting the correlation between NO_3_^−^–N and virus–host pairs. (C) Scatter plot depicting the correlation between TN and virus–host pairs. (D) Scatter plot depicting the correlation between EC and virus–host pairs. (E) Scatter plot depicting the correlation between TP and virus–host pairs. (F) Scatter plot depicting the correlation between pH and virus–host pairs. (G) Scatter plot depicting the correlation between WT and virus–host pairs. (H) Scatter plot depicting the correlation between TOC and virus–host pairs. The Spearman's correlation coefficient (*ρ*) and the associated *p* value are indicated on each plot. Only host–virus pairs with consistent correlations are shown.
**Figure S9:** Relative abundance of the identified AMGs across all six Qiangyong proglacial lake sediment samples (based on vOTU normalized contig coverage).
**Figure S10:** Number AMGs identified in the recovered viral genomes based on KEGG pathways.
**Figure S11:** The AMGs those are without homologous to the putative host. (A) The phylogenetic tree for NosZ. (B) The phylogenetic tree for AceA. (C) The phylogenetic tree for NrdA.
**Figure S12:** Virus‐encoded AMG expression. (A) The percentage of viral active AMGs involved in metabolism, genetic information processing, environmental information processing and cellular processes. (B) Distribution of viral active AMGs in 15 function categories. (C) Transcript abundance of the AMGs across all six Qiangyong proglacial lake sediment samples (based on vOTU normalized contig coverage). (D) Number of active AMGs identified in the recovered viral genomes based on KEGG pathways.

## Data Availability

The data that support the findings of this study are openly available in NCBI Sequence Read Archive (SRA) at https://www.ncbi.nlm.nih.gov/, reference number PRJNA1219359 and PRJNA1105542.
