# Supplementary material for: Diversity, Function and Activity of DNA Viruses in the Qiangyong Proglacial Lake Sediment, the Tibetan Plateau
Source: Environ Microbiol Rep. 2026 Jan 8;18(1):e70262. doi: 10.1111/1758-2229.70262 (PMC12783922; doi:10.1111/1758-2229.70262)
Supplement: Supplementary file 2 — Figure S1: The relative abundance (TPM) of the virus community. (A) Relative abundance of vOTUs across samples at class level. (B) Relative abundance of vOTUs across samples at family level. Figure S2: The transcripts abundance of the virus community. (A) Transcript abundance of viral community at the phylum, class, order and family level, respectively. (B) Transcript abundance of vOTUs across samples at class level. (C) Transcript abundance of vOTUs across samples at family level. Figure S3: Rank abundance plot showing the total relative abundance of 1336 prokaryotic MAGs across six Qiangyong proglacial lake sediment samples. Abundances were summed across sites and by phyla. Figure S4: Distribution of relative abundance of host phyla associated with viruses. Figure S5: The genomic architecture of vOTUs with selected potential anti‐defence system. Others could be found in Table S6. Figure S6: The α diversity and influencing factors of the viral community in Qiangyong proglacial lake sediments based on metagenomic. (A) The richness index of virus communities in six Qiangyong proglacial lake sediment samples. (B) The Shannon index of virus communities in six Qiangyong proglacial lake sediment samples. (C) The Evenness index of virus communities in six Qiangyong proglacial lake sediment samples. (D) Key environmental factors significantly associated with viral α‐diversity (* means p < 0.05 and ** means p < 0.01). Figure S7: Impact of biotic and abiotic factors on viral communities. (A) Distance‐based redundancy analysis (dbRDA) of bray‐Curtis dissimilarities between six proglacial lake sediments viral communities based on transcript abundance and physicochemical parameters. Vectors represent fitted environmental variables significantly correlated with dbRDA coordinates (permutation test, number of permutations = 999; . means ***p < 0.1, * means p < 0.05). (B) Procrustes rotation and substitution based on metagenomic abundance visualizes the coupling between viral and b [file EMI4-18-e70262-s001.pdf]

Supporting information to:

Diversity, function and activity of DNA viruses in the Qiangyong proglacial lake sediment, the Tibetan Plateau

Yang Zhao[1,2,3,4#], Meiling Feng[1,2,3,4#], Hongfei Chi[2,3,4], Keshao Liu[5,6], Rong Wen[1,2,3,4], Weizhen Zhang[2,3,4], Pengfei Liu[2,3,4*]

[1]College of Ecology, Lanzhou University, Lanzhou, China
[2]Center for Pan-third Pole Environment, Lanzhou University, Lanzhou, China
[3]Key Laboratory of Pan-third Pole Biogeochemical Cycling, Lanzhou, China
[4]Chayu integrated observation and research station of the Xizang Autonomous Region, Xizang, China
[5]State Key Laboratory of Tibetan Plateau Earth System, Environment and Resources (TPESER), Institute of Tibetan Plateau Research, Chinese Academy of Sciences, Beijing, China
[6]University of Chinese Academy of Sciences, Beijing, China

# These authors contribute equally.

***Correspondence author**
Pengfei Liu, (liupf@lzu.edu.cn)

This PDF file includes:

Figures S1 to S12

Supplementary figures

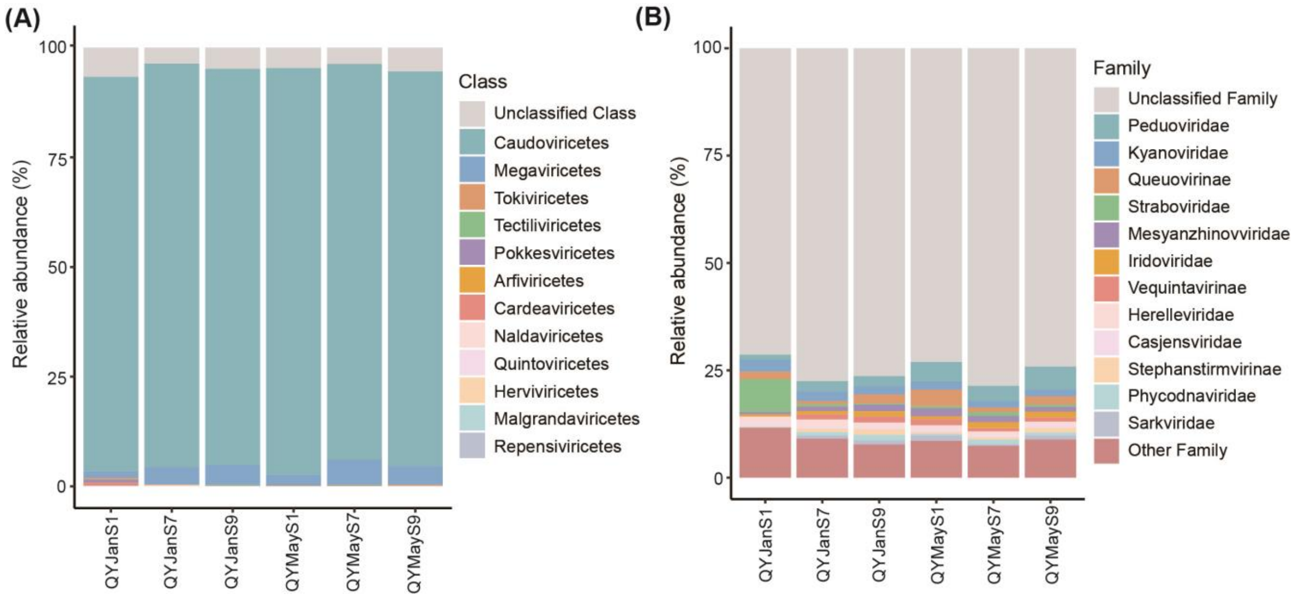

Figure S1 | The relative abundance (TPM) of the virus community. (A) Relative abundance of vOTUs across samples at class level. (B) Relative abundance of vOTUs across samples at family level.

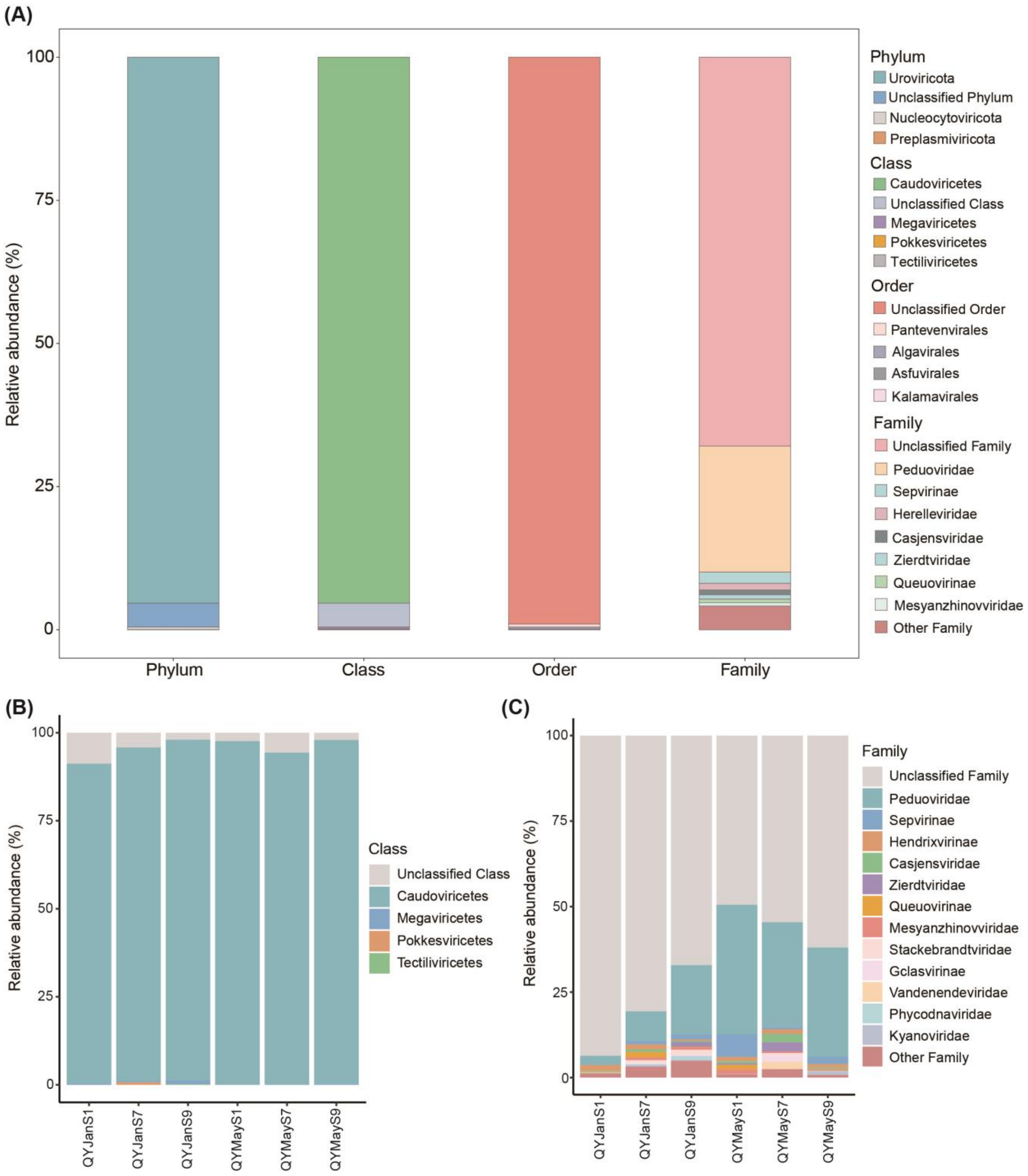

Figure S2 | The transcripts abundance of the virus community. (A) Transcript abundance of viral community at the phylum, class, order and family level, respectively. (B) Transcript abundance of vOTUs across samples at class level. (C) Transcript abundance of vOTUs across samples at family level.

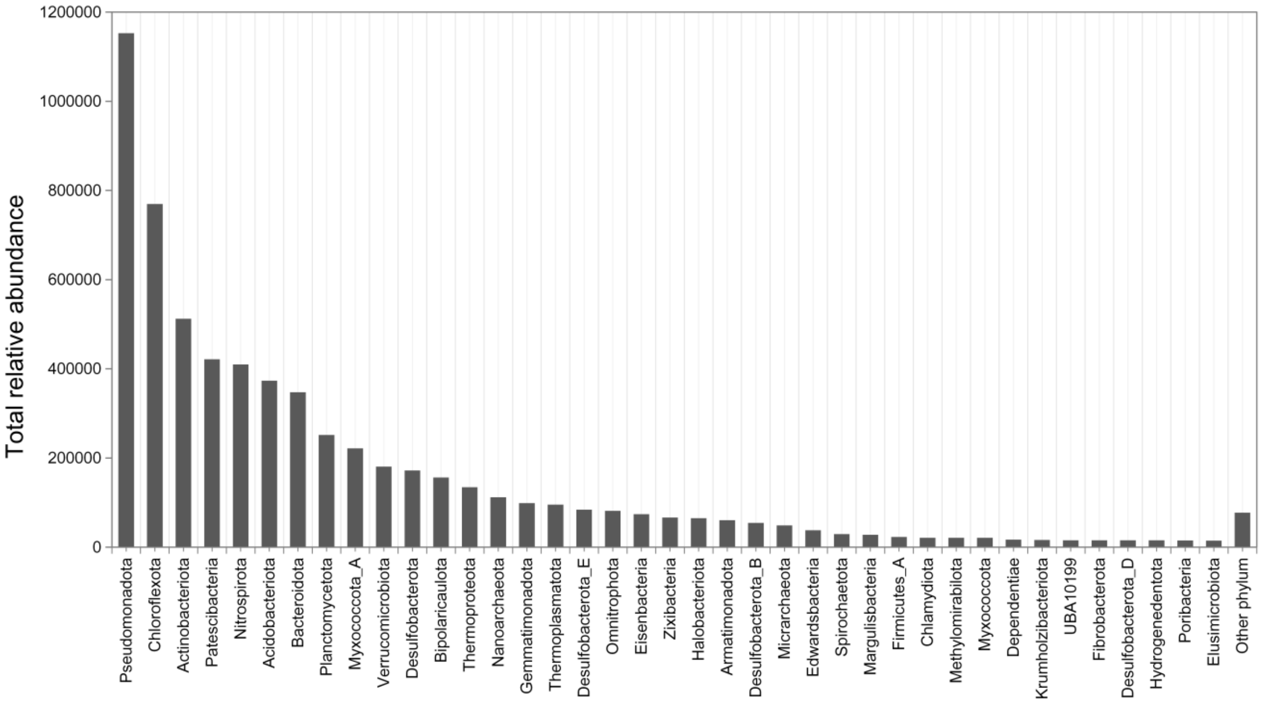

Figure S3 | Rank abundance plot showing the total relative abundance of 1336 prokaryotic MAGs across six Qiangyong proglacial lake sediment samples. Abundances were summed across sites and by phyla.

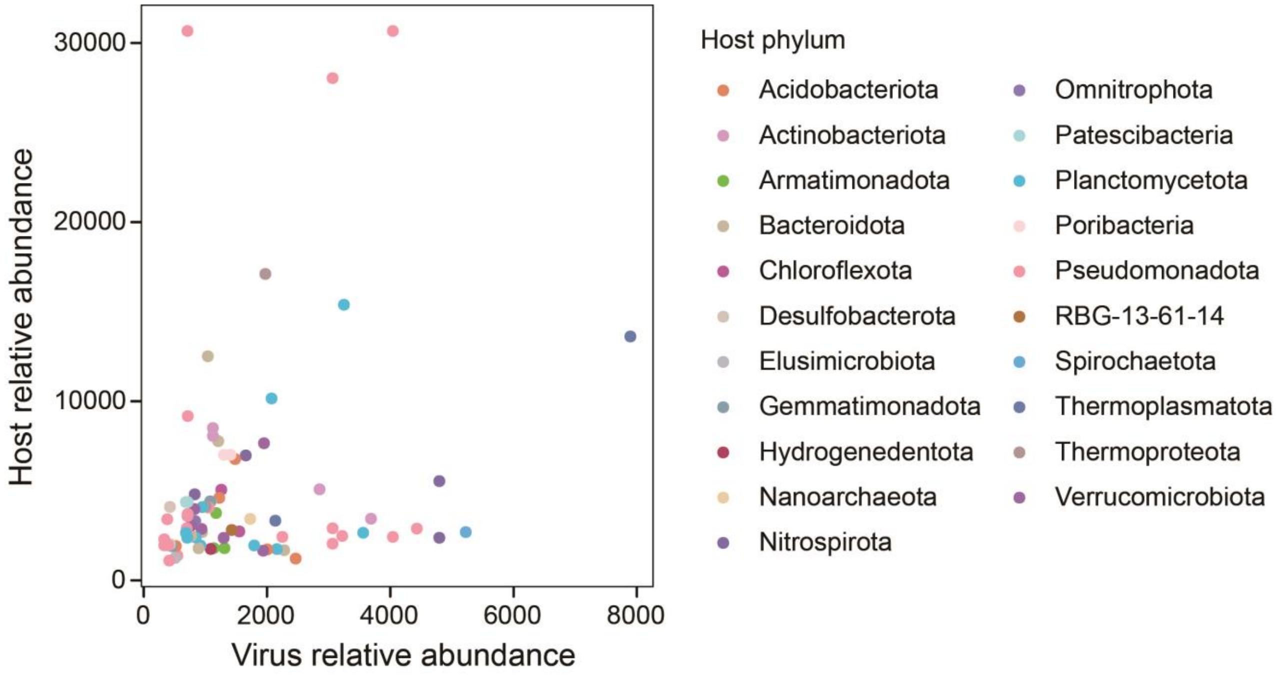

Figure S4 | Distribution of relative abundance of host phyla associated with viruses.

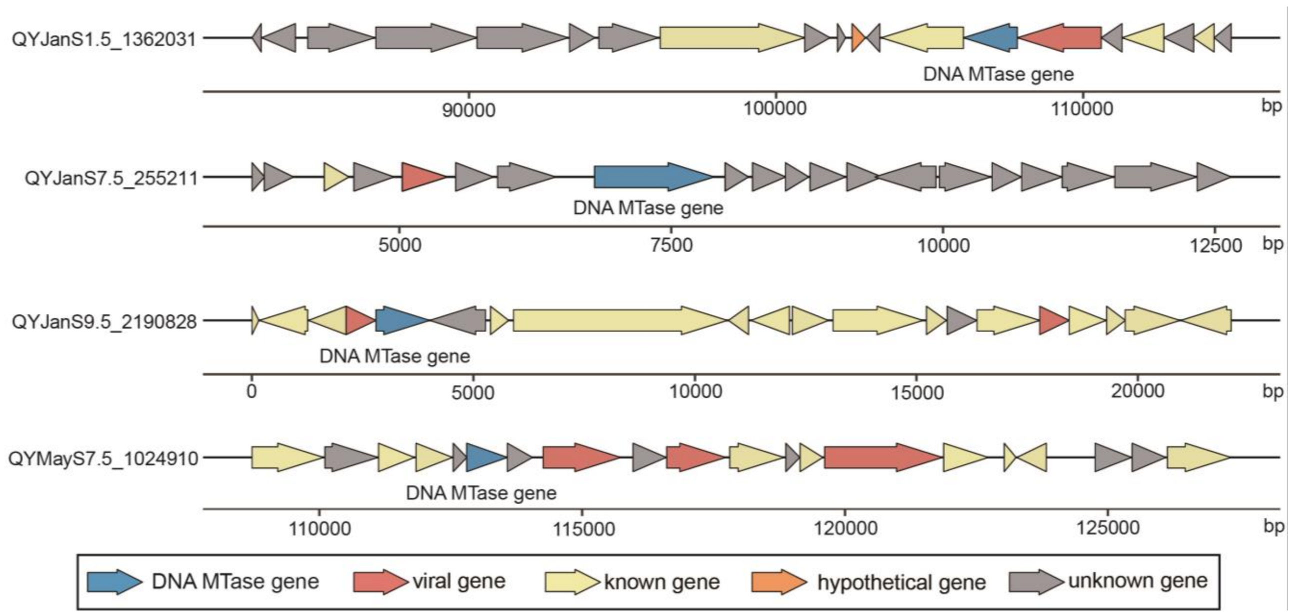

Figure S5 | The genomic architecture of vOTUs with selected potential anti-defense system. Others could be found in Table S6.

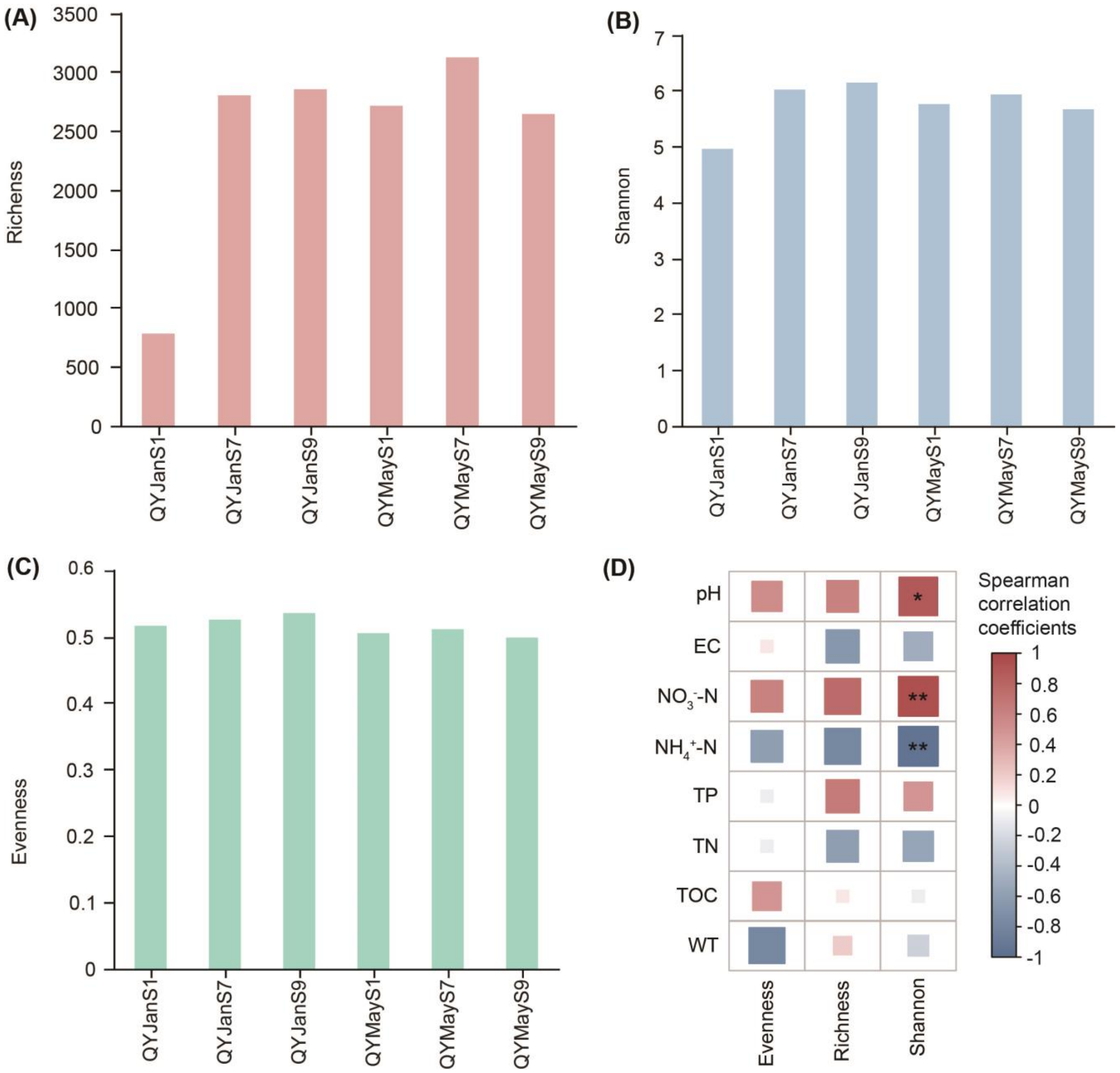

Figure S6 | The α diversity and influencing factors of the viral community in Qiangyong proglacial lake sediments based on metagenomic. (A) The richness index of virus communities in six Qiangyong proglacial lake sediment samples. (B) The Shannon index of virus communities in six Qiangyong proglacial lake sediment samples. (C) The Evenness index of virus communities in six Qiangyong proglacial lake sediment samples. (D) Key environmental factors significantly associated with viral α-diversity (* means p <0.05 and ** means p <0.01).

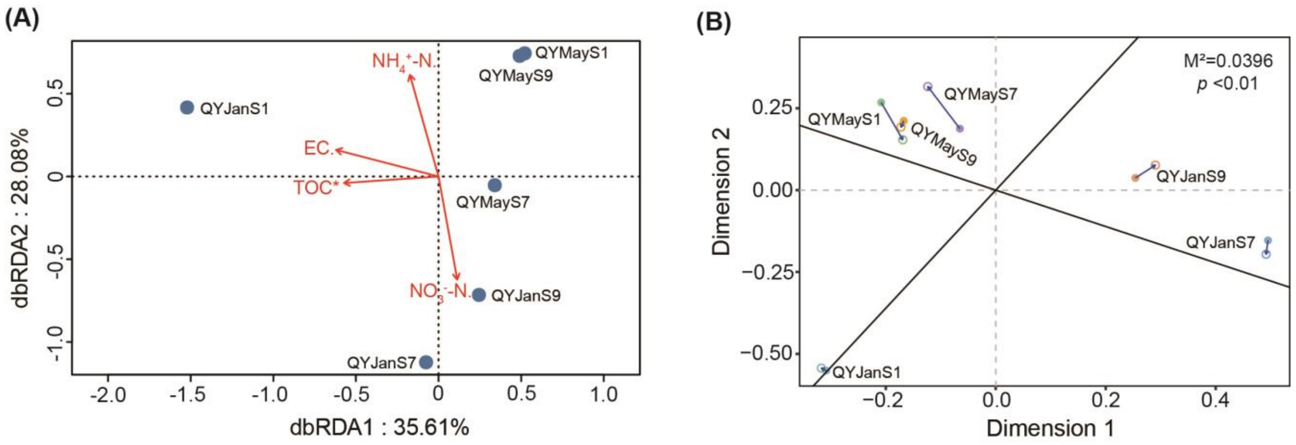

Figure S7 | Impact of biotic and abiotic factors on viral communities. (A) Distance-based redundancy analysis (dbRDA) of bray-Curtis dissimilarities between six proglacial lake sediments viral communities based on transcript abundance and physicochemical parameters. Vectors represent fitted environmental variables significantly correlated with dbRDA coordinates (permutation test, number of permutations = 999; . means p <0.1, * means p <0.05). (B) Procrustes rotation and substitution based on metagenomic abundance visualizes the coupling between viral and bacterial communities. Using Bray-Curtis distances, bacterial and viral ordinations are rescaled and connected by a line.

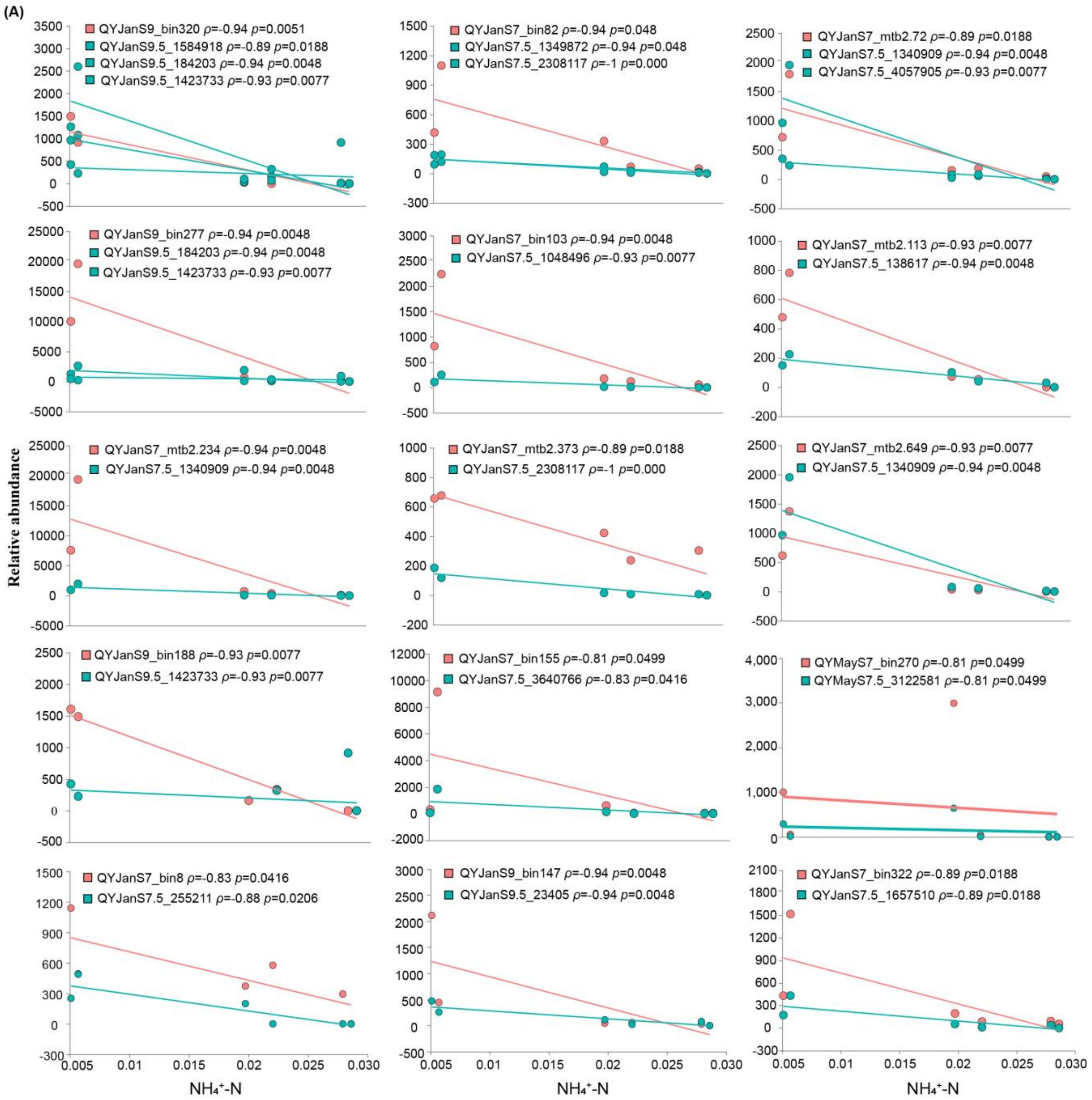

Figure S8 | Relationships between environmental factors and virus-host pairs. (A) Scatter plot depicting the correlation between NH_4^+-N and virus-host pairs. The Spearman's correlation coefficient (ρ) and the associated p-value are indicated on each plot. Only host-virus pairs with consistent correlations are shown.

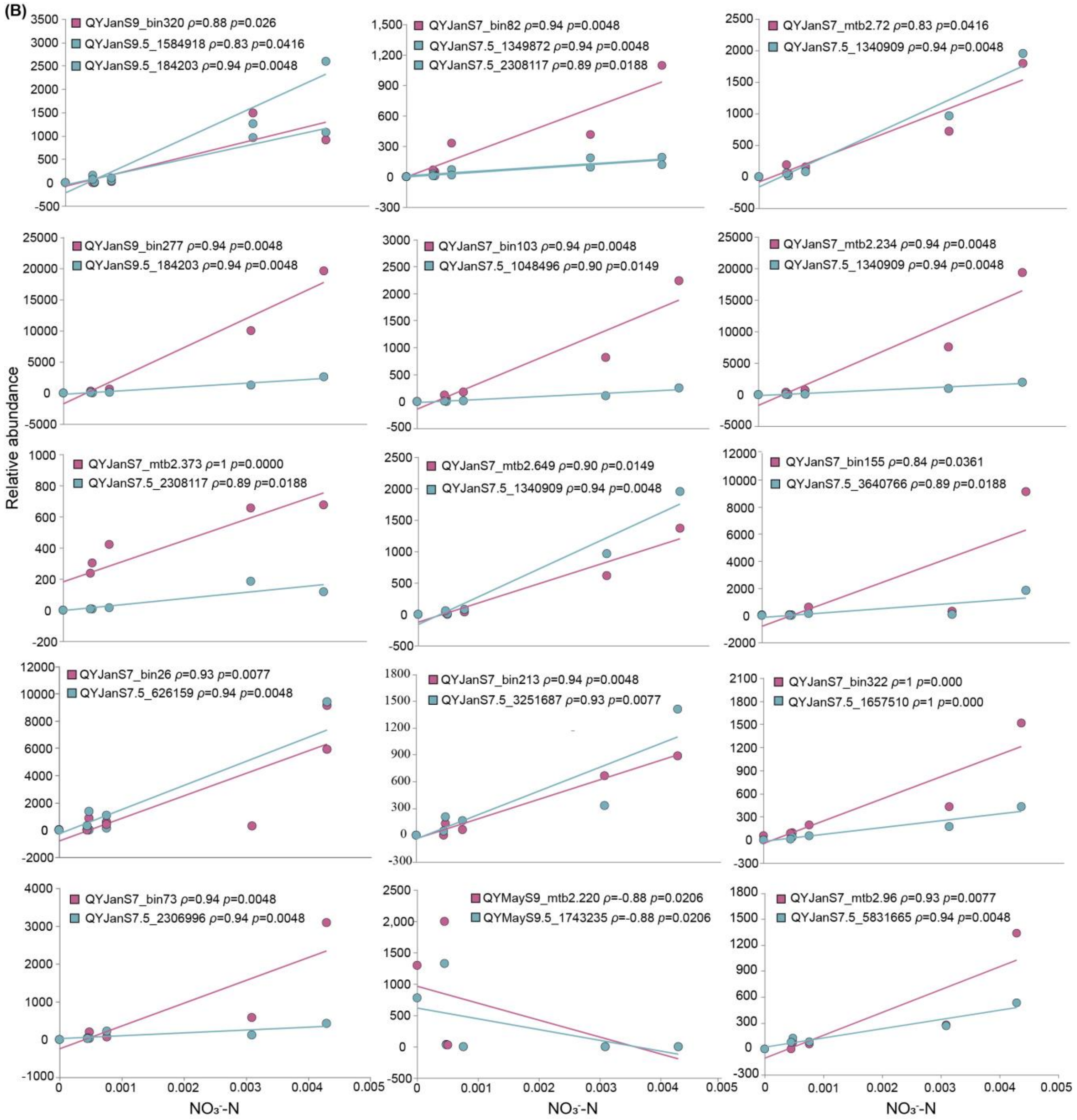

Figure S8 | Relationships between environmental factors and virus-host pairs. (B) Scatter plot depicting the correlation between NO_3^--N and virus-host pairs. The Spearman's correlation coefficient (ρ) and the associated p-value are indicated on each plot. Only host-virus pairs with consistent correlations are shown.

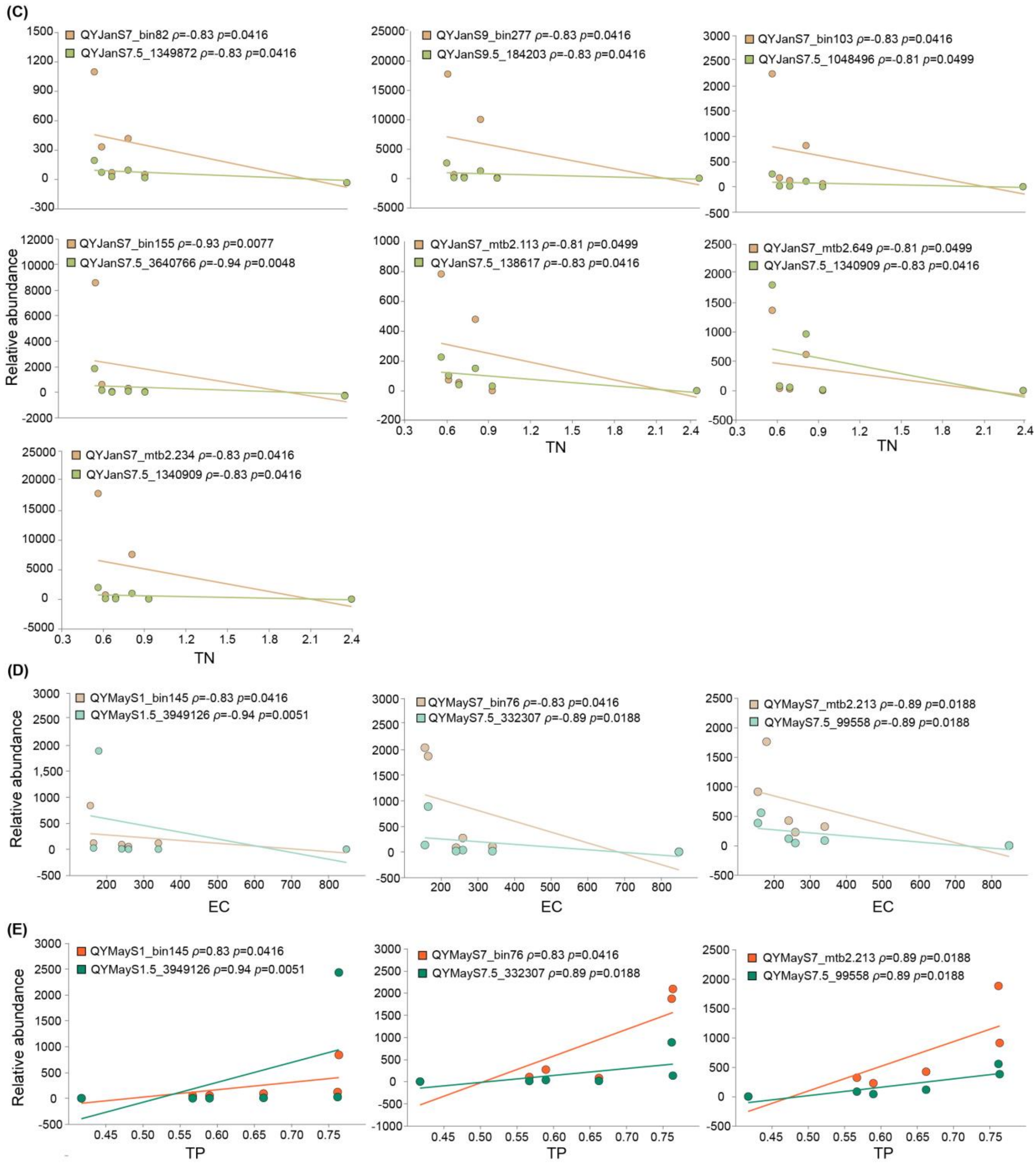

Figure S8 | Relationships between environmental factors and virus-host pairs. (C) Scatter plot depicting the correlation between TN and virus-host pairs. (D) Scatter plot depicting the correlation between EC and virus-host pairs. (E) Scatter plot depicting the correlation between TP and virus-host pairs. The Spearman's correlation coefficient (ρ) and the associated p-value are indicated on each plot. Only host-virus pairs with consistent correlations are shown.

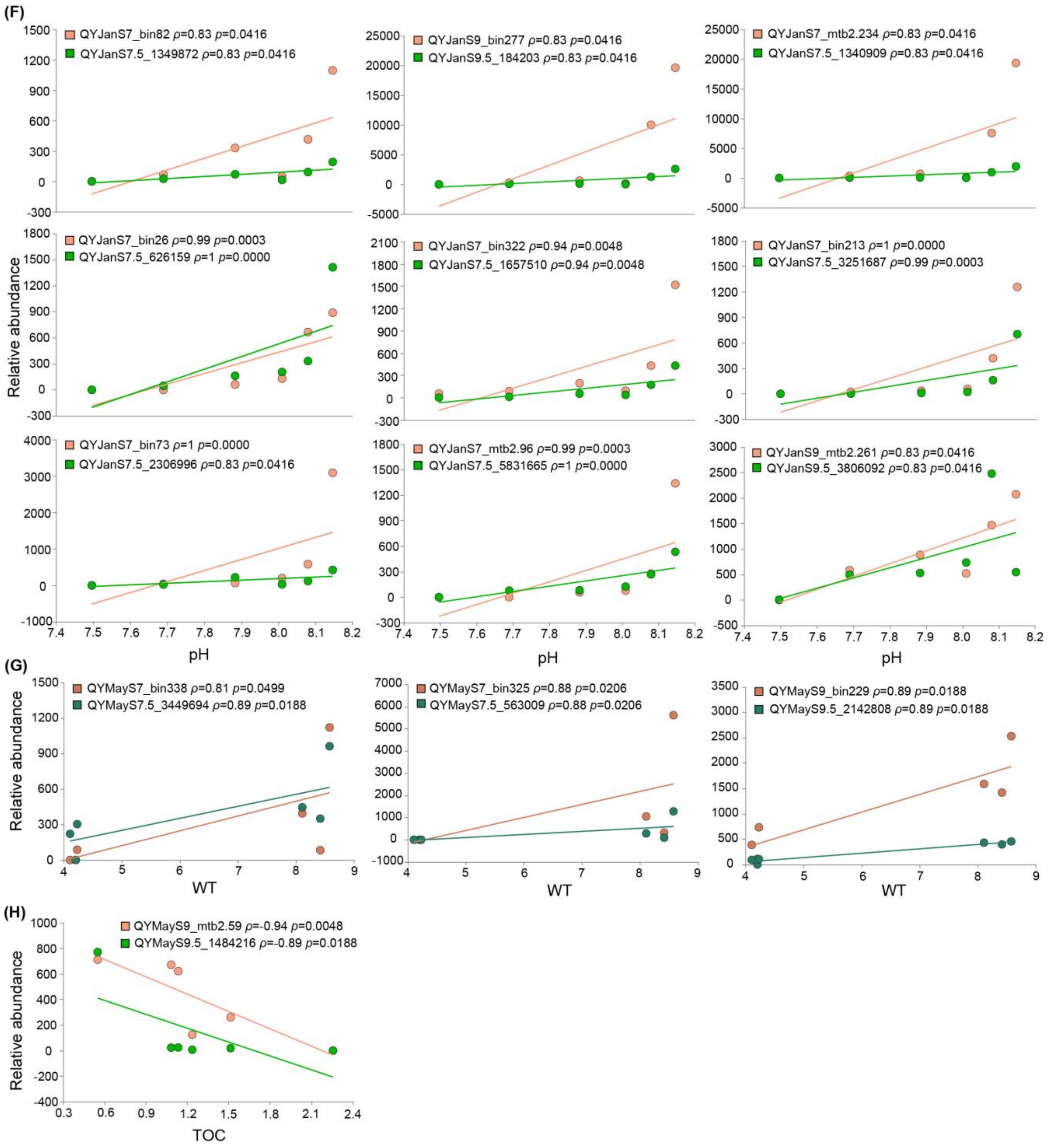

Figure S8 | Relationships between environmental factors and virus-host pairs. (F) Scatter plot depicting the correlation between pH and virus-host pairs. (G) Scatter plot depicting the correlation between WT and virus-host pairs. (H) Scatter plot depicting the correlation between TOC and virus-host pairs. The Spearman's correlation coefficient (*ρ*) and the associated *p*-value are indicated on each plot. Only host-virus pairs with consistent correlations are shown.

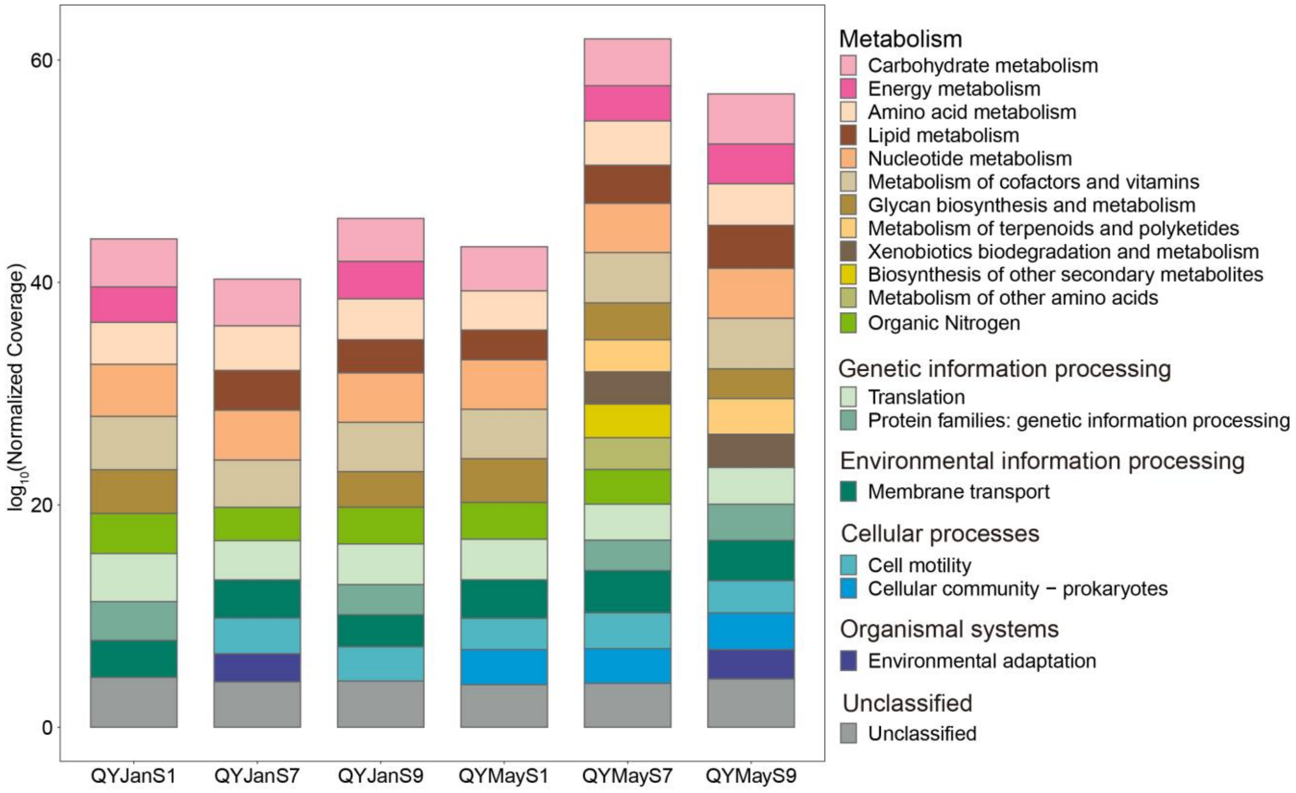

Figure S9 | Relative abundance of the identified AMGs across all six Qiangyong proglacial lake sediment samples (based on vOTU normalized contig coverage).

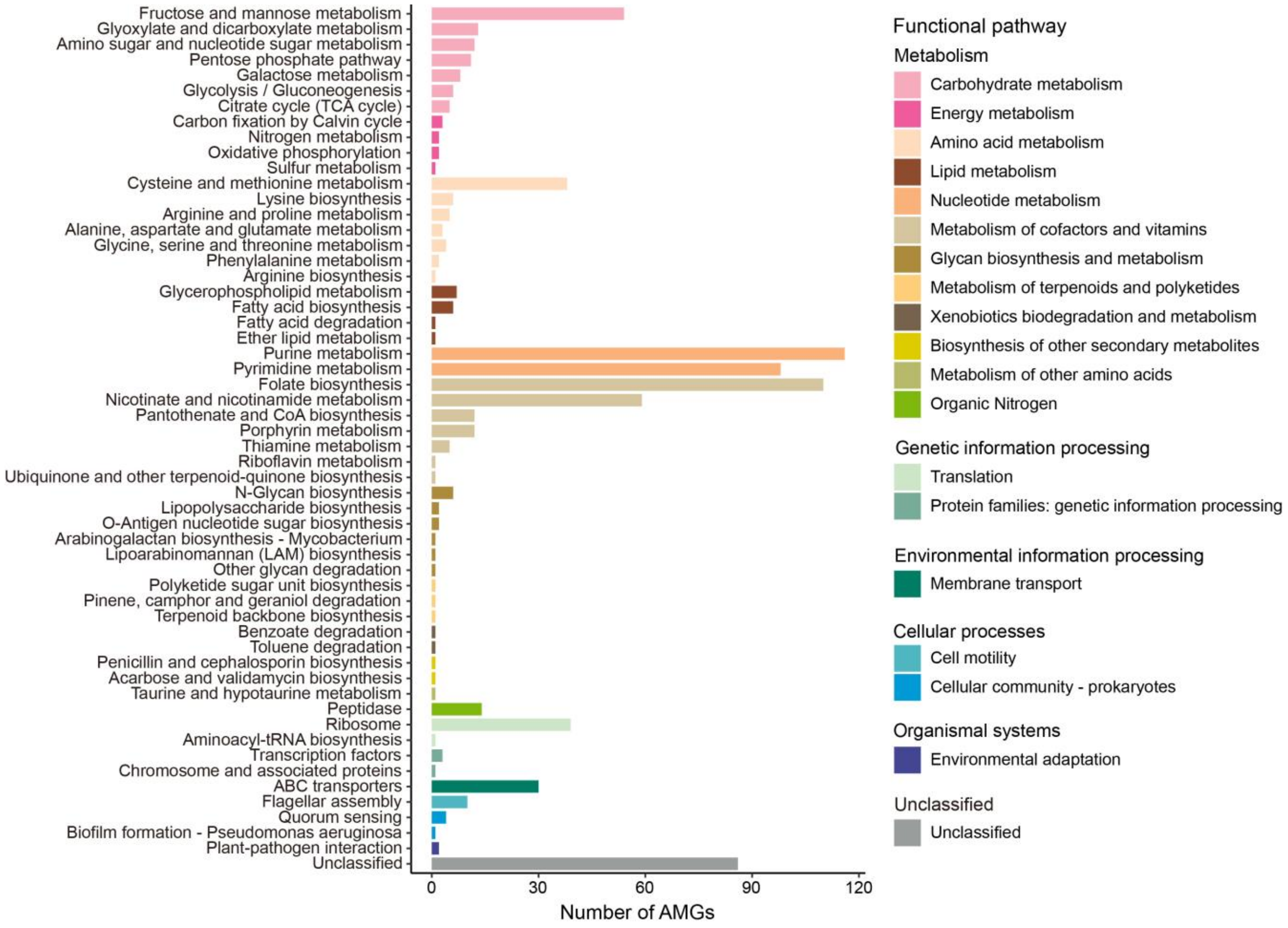

Figure S10 | Number AMGs identified in the recovered viral genomes based on KEGG pathways.

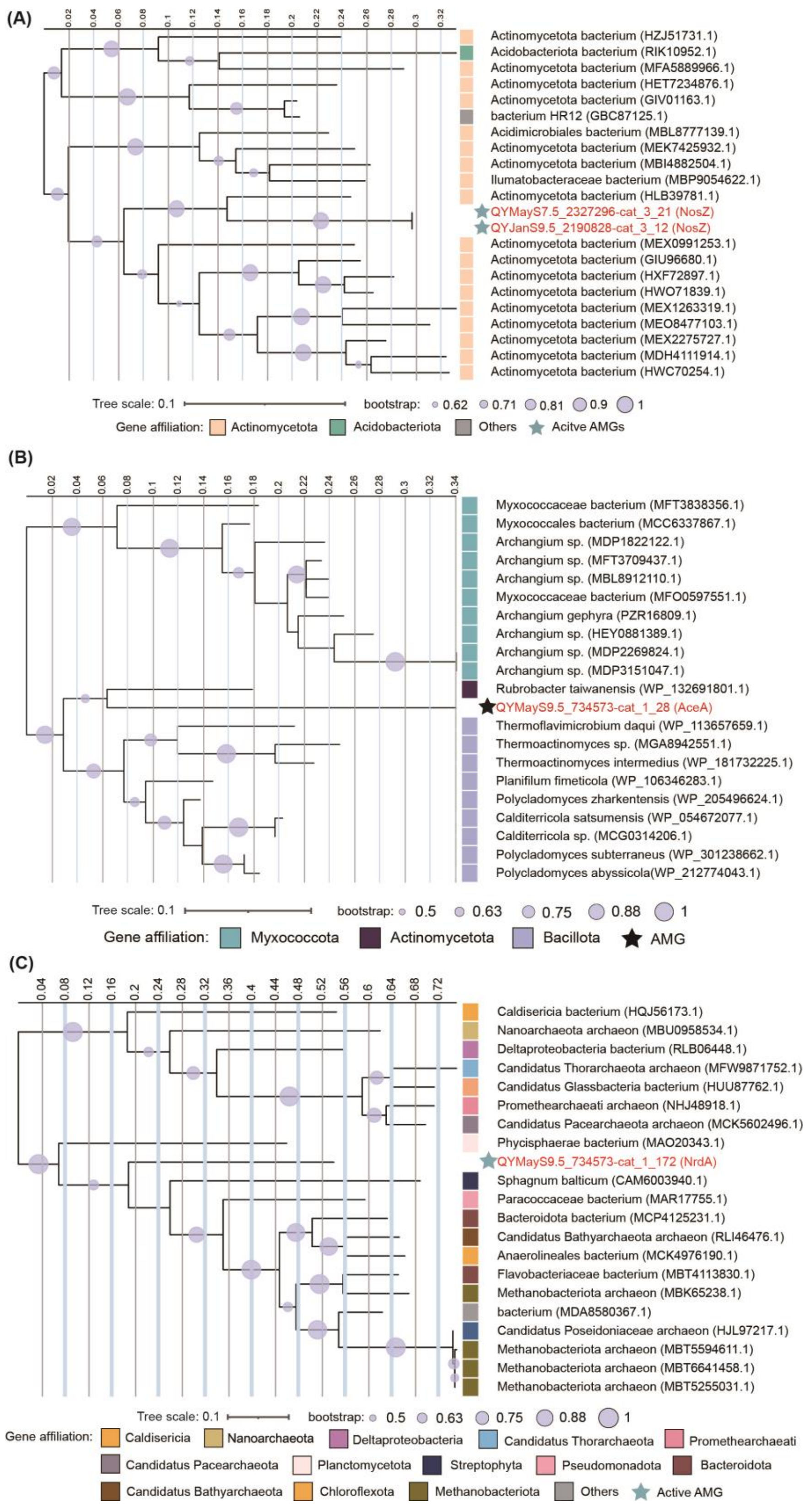

Figure S11 | The AMGs that are without homologous to the putative host. (A) The phylogenetic tree for NosZ. (B) The phylogenetic tree for AceA. (C) The phylogenetic tree for NrdA.

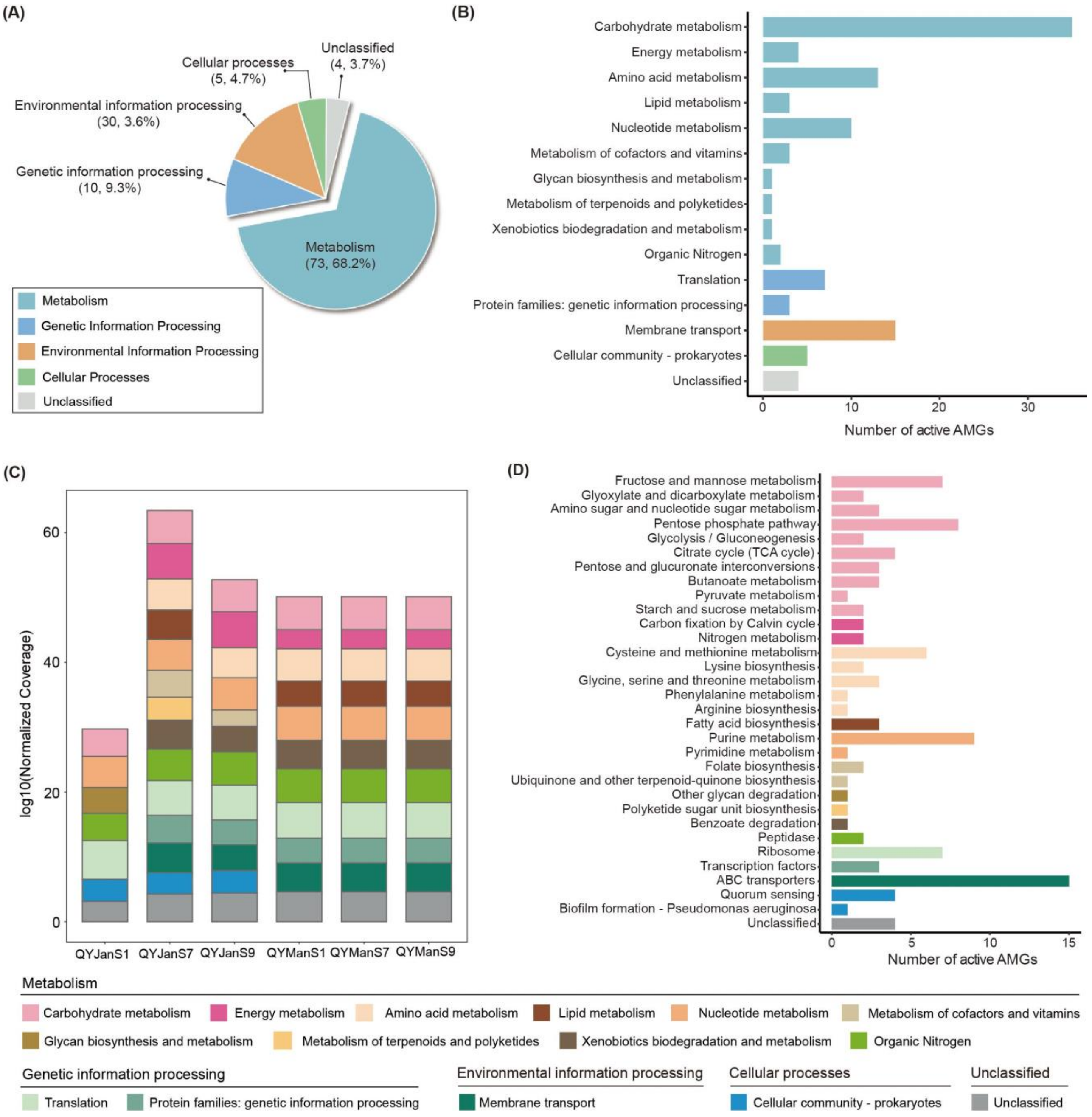

Figure S12 | Virus-encoded AMG expression. (A) The percentage of viral active AMGs involved in metabolism, genetic information processing, environmental information processing, and cellular processes. (B) Distribution of 107 viral active AMGs in 15 function categories. (C) Transcript abundance of the AMGs across all six Qiangyong proglacial lake sediment samples (based on vOTU normalized contig coverage). (D)Number of active AMGs identified in the recovered viral genomes based on KEGG pathways.
